# Do the redefined EUCAST susceptibility categories warrant adjustment of paediatric antibiotic dosages? Pragmatic physiologically based pharmacokinetic modelling of four commonly used agents

**DOI:** 10.1093/jac/dkaf463

**Published:** 2026-01-29

**Authors:** Marika A de Hoop-Sommen, Jolien J M Freriksen, Joyce E M van der Heijden, Jens Jacobs, Yvette Oosterlaan, Chantal M Staring, Shannon L van der Zeeuw, Tjitske M van der Zanden, Jan-Tom van der Bruggen, Marjolijn S W Quaak, Clementien Vermont, Tom F W Wolfs, Roger J M Brüggemann, Rick Greupink, Saskia N de Wildt

**Affiliations:** Department of Pharmacy, Pharmacology and Toxicology, Radboud University Medical Centre, Nijmegen, The Netherlands; Department of Pharmacy, Pharmacology and Toxicology, Radboud University Medical Centre, Nijmegen, The Netherlands; Department of Pharmacy, Pharmacology and Toxicology, Radboud University Medical Centre, Nijmegen, The Netherlands; Department of Pharmacy, Pharmacology and Toxicology, Radboud University Medical Centre, Nijmegen, The Netherlands; Department of Pharmacy, Pharmacology and Toxicology, Radboud University Medical Centre, Nijmegen, The Netherlands; Department of Pharmacy, Pharmacology and Toxicology, Radboud University Medical Centre, Nijmegen, The Netherlands; Department of Pharmacy, Pharmacology and Toxicology, Radboud University Medical Centre, Nijmegen, The Netherlands; Department of Hospital Pharmacy, Erasmus MC, Rotterdam, The Netherlands; Department of Pharmacy, Pharmacology and Toxicology, Radboud University Medical Centre, Nijmegen, The Netherlands; Department of Paediatric and Neonatal Intensive Care, Erasmus MC-Sophia Children’s Hospital, Rotterdam, The Netherlands; Dutch Knowledge Centre Pharmacotherapy for Children, The Hague, The Netherlands; Department of Medical Microbiology, University Medical Centre Utrecht, Utrecht, The Netherlands; Department of Paediatrics, Dijklander Ziekenhuis, Hoorn, The Netherlands; Department of Paediatrics, Division of Infectious Diseases and Immunology, Erasmus MC University Medical Centre-Sophia Children's Hospital, Rotterdam, The Netherlands; Department of Paediatrics, Division of Infectious Diseases and Immunology, Erasmus MC University Medical Centre-Sophia Children's Hospital, Rotterdam, The Netherlands; Department of Paediatrics, Wilhelmina Children's Hospital, Utrecht, The Netherlands; Department of Pharmacy, Pharmacology and Toxicology, Radboud University Medical Centre, Nijmegen, The Netherlands; Radboud Institute for Medical Innovation, Radboud University Medical Centre, Nijmegen, The Netherlands; Department of Pharmacy, Pharmacology and Toxicology, Radboud University Medical Centre, Nijmegen, The Netherlands; Department of Pharmacy, Pharmacology and Toxicology, Radboud University Medical Centre, Nijmegen, The Netherlands; Department of Paediatric and Neonatal Intensive Care, Erasmus MC-Sophia Children’s Hospital, Rotterdam, The Netherlands; Dutch Knowledge Centre Pharmacotherapy for Children, The Hague, The Netherlands; Department of Intensive Care, Radboud University Medical Centre, Nijmegen, The Netherlands

## Abstract

**Background and objective:**

With the redefinition of the EUCAST ‘I’ susceptibility category, from ‘intermediate’ to ‘susceptible, increased exposure’, the focus is now to use higher doses to treat infections of this category. These higher dosages in adults provided by EUCAST are fixed, yet it is uncertain whether a similar increase should apply to paediatric doses. We aimed to compare antibiotic exposure in adults and children, using pragmatic physiologically based pharmacokinetic (PBPK) modelling and simulation to evaluate the need for dose increases in children for the ‘I’ susceptibility category micro-organisms.

**Methods:**

For amoxicillin, ceftazidime, cefuroxime and ciprofloxacin, we used existing PBPK models and verified them with published adult and paediatric pharmacokinetic data. Then, the adult EUCAST category ‘I’ doses and a wide paediatric dosage range was simulated. We compared AUC values as a surrogate for antibiotic exposure. In addition, simulations were performed to assess the PTA of the adult and paediatric doses for specific drug–micro-organism combinations.

**Results:**

Model verification proved successful for all antibiotics. Simulations showed that antibiotic plasma exposure increases with decreasing age using current paediatric doses. Simulating doses for neonates and infants resulted in substantially higher AUCs compared with adults receiving the EUCAST ‘I’ dose. Simulations showed that PTA is highly variable with age and can be poor in case of less susceptible micro-organisms.

**Conclusions:**

In contrast to adults, there is no need to increase the currently recommended paediatric dosages for the tested antibiotics. At the same time, further simulations showed that the PTA varies by drug-micro-organism combination and age, providing a potential opportunity to tailor doses to individual patients.

## Introduction

Since 2002, the EUCAST has used three definitions to categorize the susceptibility of micro-organisms to an antimicrobial agent, i.e. ‘susceptible’ (S), ‘intermediate’ (I) and ‘resistant’ (R). While the use of the ‘S’ and ‘R’ categories was clear, the use of the ‘I’ category contained many uncertainties and was often wrongly considered as ‘another resistant’ category. Consequently, the naming and definitions of the categories were modified in 2019 to ‘susceptible, standard dosing regimen’ (S), ‘susceptible, increased exposure’ (I) and ‘resistant’ (R), leaving the abbreviations the same. Essentially, the meaning of the categories has not changed, but they have been worded more clearly to avoid misunderstandings.^1^ The new definitions reflect the need for tailored plasma exposure, which should be achieved with dosages equivalent to those in the EUCAST clinical breakpoint tables, recommending increased exposure for category ‘I’ infections.^2^ These EUCAST dosages primarily serve to inform about the level of exposure, but they are fixed adult doses that cannot be used for all paediatric patients. Moreover, it is questionable whether a doubled dose in adults leads to the same effect as a doubled dose in children, given the differences in pharmacokinetics (PK) and pharmacodynamics (PD) between the two patient populations. So, for now, it remains unclear how the increased dosages of the ‘I’ category should be translated to paediatric dosages.

To address this information gap and to provide dosing guidance, a joint task force by the EUCAST and the European Society for Paediatric Infectious Diseases aimed to establish both ‘S’ and ‘I’ dosing recommendations for children beyond the neonatal age.^3^ Lowest and highest dose recommendations were compiled from four national paediatric dosing guidelines for the most frequently used antibiotics, and feedback was solicited from national antimicrobial susceptibility testing teams from across Europe. In addition, paediatric healthcare professionals were invited to participate in a survey to test the extent to which the dosing recommendations represent clinical practice.^3^

In addition to the efforts of the task force, this information gap can also be addressed from a pharmacokinetic perspective. Pharmacokinetic modelling and simulation can be used to simulate dosing scenarios to select the most optimal ones. An attractive approach in this context is physiologically based pharmacokinetic (PBPK) modelling. PBPK models mathematically describe the human body using multiple compartments that represent human organs and tissues, which are connected by blood flow. Physiological characteristics are combined with drug-specific data to predict PK. A paediatric PBPK model also includes age-related developmental changes (e.g. organ growth, renal function, and ontogeny of drug-metabolizing enzymes), making it well suited to predict drug exposure in this population. Even in the context of limited pharmacokinetic data, which is often the case in paediatrics, these models can provide trustworthy dose simulations when adequately verified.

Today, PBPK models have been established for >300 drugs to describe pharmacokinetics and simulate doses, for example, for adult and paediatric populations and for drug–drug interactions.^4^ These drug-specific models can pragmatically be reused by connecting them to a paediatric population model to simulate age-appropriate dosages.^5^

In this study, we aimed to use this pragmatic PBPK modelling approach by simulating drug exposure in adults, children and term neonates with adult EUCAST and several paediatric doses. We hypothesized that if plasma exposure in children is similar to that in adults, paediatric dosages do not warrant adjustment. In addition, we aimed to explore the PTAs of both adult and paediatric dosages for frequent drug–micro-organism combinations of the ‘I’ category.

## Methods

For this study, four antibiotics were chosen as a proof-of-concept from the WHO model list of essential medicines for children^6^ for which (i) EUCAST recommends increased dosing in category ‘I’ infections and (ii) a PBPK model is already available, i.e. amoxicillin (AMX), ceftazidime (CAZ), cefuroxime (CXM) and ciprofloxacin (CIP). The study comprised three parts: first, we selected existing PBPK models for AMX, CAZ, CXM and CIP, and verified them with published adult and paediatric PK data. We then simulated the adult EUCAST doses and the low and high doses reported by the task force and compared the predicted AUCs between adults and different paediatric age groups. Finally, we assessed the PTAs of the simulated dosages for frequent drug-micro-organism combinations of the ‘I’ category.

### Model selection and verification

The PBPK modelling platform Simcyp^™^ (Certara, Sheffield, UK) was used for all simulations. Default Simcyp populations were used; for adults we used the ‘Sim-healthy volunteer’ population or the ‘Sim-NEurCaucasian’ in case patients above 65 years of age were included. For children, the ‘Sim-Paediatric’ population was used (Figure S1, available as Supplementary data at *JAC* Online). Input parameters for the PBPK models of AMX, CAZ, CXM and CIP can be found in Table S1.^7–9^

Model verification of CAZ was previously performed in Simcyp version 20 and published by van der Heijden *et al.*^10^ For the verification of the AMX, CXM and CIP models and for all further simulations, Simcyp version 22 was used. We used clinical PK data from the literature (PubMed database), retrieved using standardized search queries (Supplementary Data Section 2, Table S2). These clinical PK studies (Tables S3a–S5b) were replicated using a similar virtual trial design as the original PK study (e.g. age range, proportion of females, etc.). Ten virtual PK trials, each with 10 virtual subjects, were conducted. To assess the accuracy of the model predictions, we compared predicted plasma concentration–time profiles with observed data. To this end, a visual predictive check (VPC) was conducted, and predicted-to-observed (P/O) PK parameter ratios were calculated for the reported PK parameters.^5^ PK parameter ratios within a 2-fold range were considered acceptable, with data being assessed for broader trends rather than isolated discrepancies.^11^

### Model simulations; drug exposure

To compare drug exposure between adults and paediatric patients, we simulated several adult and paediatric dosages (Table 1). Both the low and high dose defined by the task force were simulated across nine paediatric age groups (i.e. 1, 2, 3, 6 and 9 months, 1–2, 2–6, 6–12 and 12–18 years).^3^ As these doses were intended for children beyond neonatal age, we added simulations of neonatal dosing recommendations from the Dutch Paediatric Formulary (DPF) in five additional age groups (i.e. 1 day, 1, 2, 3 and 4 weeks postnatal age).^17^ In addition, the DPF recommends a doubled CXM dose compared with the task force for children from the age of one month, which was therefore also included in the simulations. The predicted 24-hour AUC at steady state was used as a surrogate for antibiotic exposure. Owing to the short half-lives of these drugs, steady state is reached within 24 hours for all four drugs, hence the duration of each simulation was set at 48 hours. AMX and CAZ were infused over 30 minutes, and CXM and CIP over 60 minutes. The ‘redefine subjects over time’ function in Simcyp, allowing for anatomical and physiological growth of the virtual subjects during the simulation period, could not be used because of incompatibility with the Mechanistic Kidney Model and/or the unbound plasma concentrations that were needed for analysis. However, as the duration of each simulation was only 48 hours, it is expected that the effect of the ‘redefine subjects over time’ function on model simulations would be negligible.

**Table 1.** Neonatal, paediatric and adult dosages

(a) Amoxicillin (AMX)	IV	(b) Amoxicillin (AMX)	PO
Age	Dose	Age	Dose
Adult low dose:	1 g q8h	Adult low dose:	0.5 g q8h
Adult high dose:	2 g q4h	Adult high dose:	1 g q8h
Low paediatric dose:		Low paediatric dose:	
1 mo–18 y	20 mg/kg q8h,	1 mo–18 y	15 mg/kg q8h,
	max 12 g/day		max 3 g/day
High paediatric dose:		High paediatric dose:	
<1 wk, ≥2 kg	25 mg/kg q8h	<1 mo	30 mg/kg q8h
1–4 wk, ≥2 kg	25 mg/kg q6h	1 mo–18 y, <40 kg	30 mg/kg q8h,
1 mo–18 y	50 mg/kg q6h,		max 3 g/day
	max 12 g/day	1 mo–18 y, ≥40 kg	0.5–1 g q8h,
			max 6 g/day
(c) Ceftazidime (CAZ)	IV	(d) Cefuroxime (CXM)	IV
Age	Dose	Age	Dose
Adult low dose:	1 g q8h	Adult low dose:	0.75 g q8h
Adult high dose:	1 g q4h	Adult high dose:	1.5 g q8h
Low paediatric dose:		Low paediatric dose:	
1 mo–18 y	25 mg/kg q8h,	1 mo–18 y	20 mg/kg q8h,
	max 6 g/day		max 4.5 g/day
High paediatric dose:		High paediatric dose:	
<1 wk, ≥2 kg	50 mg/kg q12h	<1 wk, ≥2 kg	25 mg/kg q8h
1–4 wk, ≥2 kg	50 mg/kg q8h	1–4 wk, ≥2 kg	33.3 mg/kg q8h
1 mo–18 y	50 mg/kg q8h,	1 mo–18 y	25 mg/kg q6h,
	max 6 g/day		max 6 g/day
		DPF dose:	
		1 mo–18 y	50 mg/kg q6h,
			max 6 g/day
(e) Ciprofloxacin (CIP)	IV	(f) Ciprofloxacin (CIP)	PO
Age	Dose	Age	Dose
Adult low dose:	0.4 g q12h	Adult low dose:	0.5 g q12h
Adult high dose:	0.4 g q8h	Adult high dose:	0.75 g q12h
Low paediatric dose:		Low paediatric dose:	
1 mo–18 y	10 mg/kg q12h,	1 mo–18 y	10 mg/kg q12h,
	max 0.8 g/day		max 1 g/day
High paediatric dose:		High paediatric dose:	
<1 mo	12.5 mg/kg q12h	1 mo–18 y	20 mg/kg q12h,
1 mo–18 y	10 mg/kg q8h,		max 1.5 g/day
	max 1.2 g/day		

Dosages for neonates and the maximum paediatric doses were taken from the DPF indications ‘severe infections’.^12–15^ Low and high paediatric dosages are the task force dosages and the adult dosages are from the EUCAST breakpoint tables.^16^ The high task force doses generally correspond to DPF doses, except for CXM, where an aberrant (higher) DPF dose is added.^2,3,12–15^ Note that the underlined dosages, originating from the DPF, indicate that the paediatric maximum dose is higher than the adult dose.

PO, oral; q, every; wk, week; y, year.

### Model simulations; PTA

All model simulations to determine the PTAs for each dose were performed in Simcyp version 22. For each antibiotic, target attainment was assessed for one relevant micro-organism from wild-type category ‘I’ (Table 2), and for worst-case scenarios such as critical illness, the drug-specific breakpoint was selected for these micro-organisms. Pharmacodynamic (PD) targets were retrieved from the EUCAST website (Table 2).^2,36^ However, the cut-off values for these PD targets [e.g. percentage of time that the free (or unbound) plasma concentration is above the MIC (%*f*T > MIC)] differed largely between EUCAST rationale documents and the scientific literature. The EUCAST based its cut-off values mainly on animal data, while emerging clinical data have shown that higher targets may be required, especially for critically ill patients.^23,31,37,38^ To illustrate the impact of these different cut-off values, we assessed the PTA for both the EUCAST cut-off values and the most commonly used cut-off values as reported in literature (Table 2). The PTA was calculated for all simulated dosages (Table 1), using unbound plasma concentrations for AMX, CAZ and CXM, and total plasma concentrations for CIP, as the CIP therapeutic target was interpreted as such.^33,39^ A PTA >90% is typically considered sufficient.^40^

**Table 2.** Drug-micro-organism combinations with corresponding PD targets

				Cut-off value PD target:		
Drug	Micro-organism	Breakpoint	PD target^a^	EUCAST	Literature	Ref.
Amoxicillin	*Haemophilus influenzae*	2 mg/L	%*f*T > MIC	40%	IV: 50–70%^c^	^18–22^
					PO: 50%	^22–25^
Ceftazidime	*Pseudomonas aeruginosa*	8 mg/L	%*f*T > MIC	65%	100%	^26–30^
Cefuroxime	*Escherichia coli*	8 mg/L	%*f*T > MIC	65%^d^	100%	^29,31,32^
Ciprofloxacin	*Pseudomonas aeruginosa*	0.5 mg/L	AUC/MIC	≥90^b^	≥125	^33–35^

%*f*T > MIC, percentage of time that the free plasma concentration is above the MIC; PD, pharmacodynamic; PopPK, population pharmacokinetic.

^a^For MIC, the breakpoint was used.^41^

^b^EUCAST reported an unbound *f*AUC/MIC ratio of 72, along with 20%–30% protein binding. This is recalculated to a total AUC/MIC ratio range of 90–103. The lower ratio value was chosen, as the 20% protein binding was in better agreement with the protein binding in the PBPK model (i.e. 21.4%).

^c^Published PopPK studies used several targets, up to 100%*f*T > MIC. As PTAs for many age groups are less than 90% aiming at 70%*f*T > MIC, we did not include these higher targets.

^d^EUCAST rationale document for cefuroxime did only report a percentage for bacteriostasis. As this percentage is in the same range as EUCAST reported for bacteriostasis by ceftazidime (also a cephalosporin), we took the percentage as reported for ceftazidime.^29^

## Results

### Model verification

**Figure 1.**
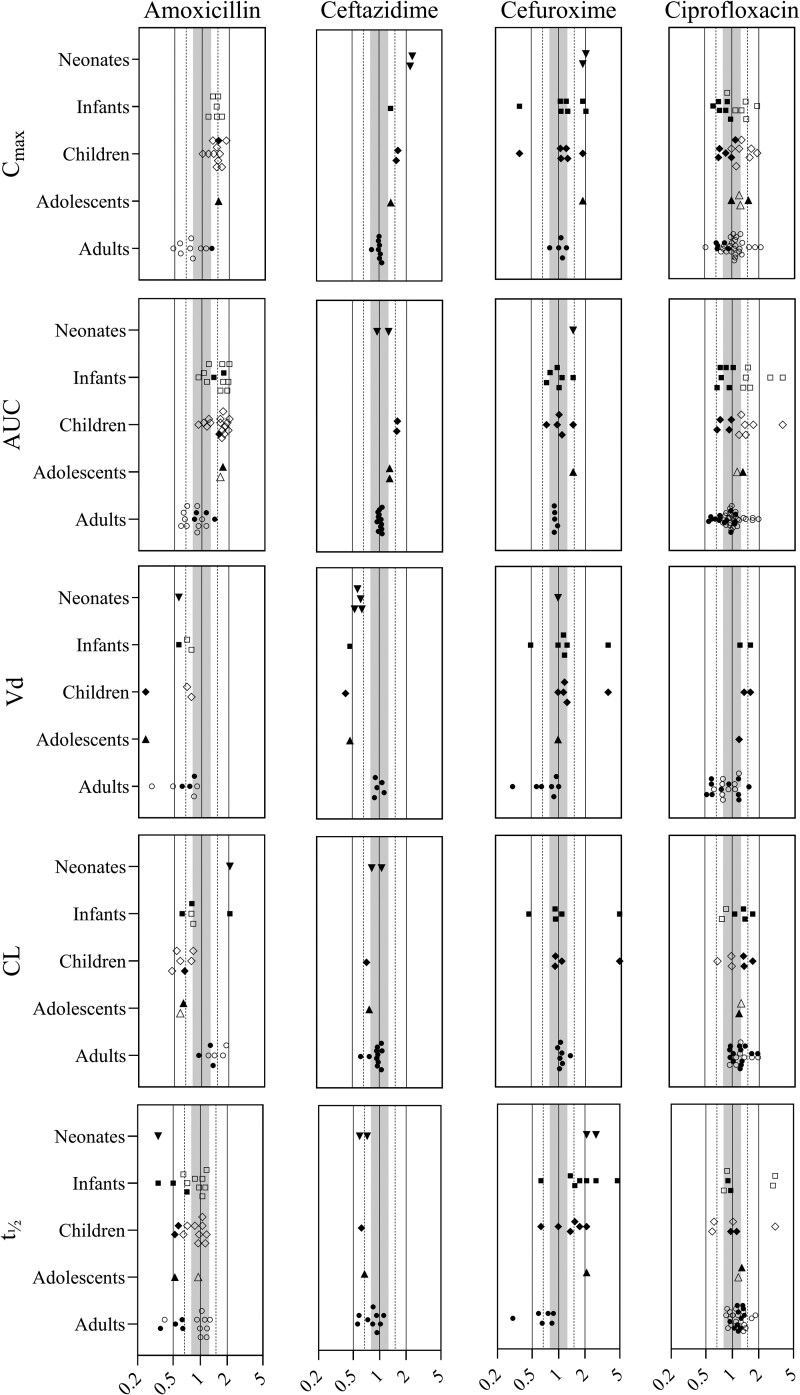
Predicted-to-observed PK parameter ratios. Age groups are: term neonates 0–28 days postnatal age, 1–24-month-old infants, 2–12-year-old children and 12–18-year-old adolescents. The middle solid line is the unity line, the outer solid lines, dotted lines and shaded area indicate the 2-fold, 1.5-fold and 1.25-fold ranges, respectively. Open symbols represent oral administration, closed symbols represent IV administration. Abbreviations: CL, clearance; C_max_, maximum plasma concentration; IV, intravenous; t½, elimination half-life; Vd, volume of distribution.

The PBPK models for AMX, CAZ and CIP were used in their published form, without changes, as they accurately predicted PK in adults and children, with 90%, 96% and 97% of all P/O PK parameter ratios falling within the acceptable 2-fold range, respectively (Figure 1, Table S6a for P/O PK parameter ratios, Figures S2a–d and S4a–g for VPCs). For CXM, the model performed well in adults, but simulations of PK in paediatric subjects were less accurate, as the volume of distribution (*V*_d_) and clearance (CL) were both underpredicted. To use the model for further predictions, we had to move away from our ‘pragmatic approach’ for this particular drug. After changing the *V*_d_ by adjusting the *K*_p_ scalar (based on clinically observed *V*_d_ values), and deactivating the Mechanistic Kidney Model (using overall renal clearance instead), 77% of paediatric model predictions were within 2-fold (Table S1 for input parameters, Figure 1, Table S6a for P/O PK parameter ratios, and Figure S3a–c for VPCs). Deactivation of the Mechanistic Kidney Model also improved predictions in adults, so we applied this change in the adult model as well.

### Model simulation; drug exposure

**Figure 2.**
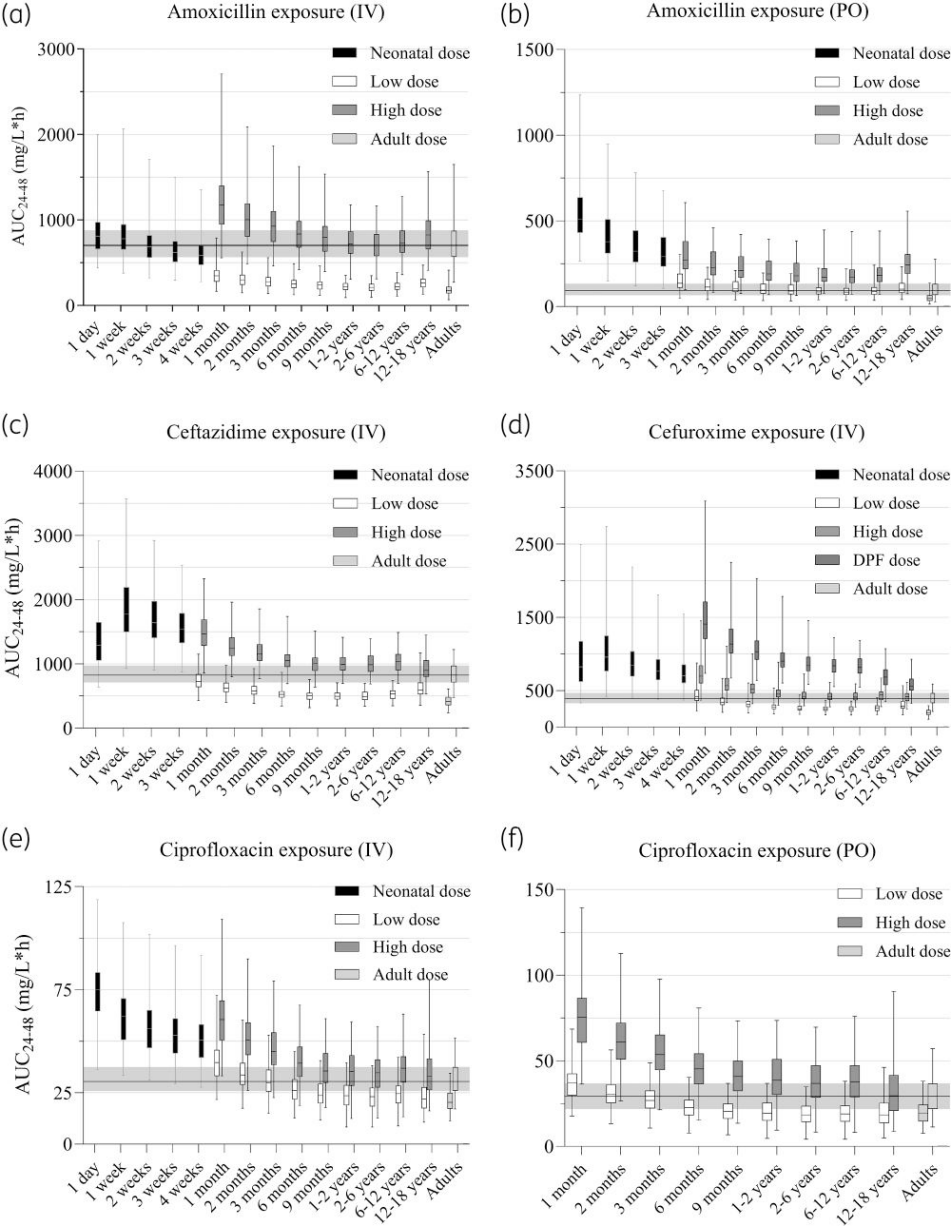
Daily antibiotic exposure in neonates, children and adults at steady state. The simulated dosages can be found in Table 1. The boxes represent the IQR around the median, the whiskers are minimum and maximum of the predicted AUCs. The shaded area shows the predicted IQR of the adult high dose exposure. For older children, the maximum doses (as reported in Table 1) are applied in case the mg/kg dose exceeds the maximum dose. Abbreviations: AUC_24-48_, AUC from 24 to 48 hours after the first dose; IQR, interquartile range; IV, intravenous; mo, months; PO, oral; wk, weeks; y, years.

Figure 2 shows the predicted antibiotic exposure of all dosages as described in Table 1. Some consistency in exposure across the age range appears to be lacking. Moreover, when the median exposure of the high paediatric doses is compared with that of the high adult doses, it is higher in all paediatric age groups than in adults. This is also true for the low dose, with two exceptions, where the median oral CIP AUC_24-48_ in adults at 19.5 mg/L*h is only slightly higher than that of 2–6 and 12–18-year-olds at 18.38 and 18.42 mg/L*h, respectively. For most drugs, the age group with the lowest AUC is that of 2–6 years. In case of ciprofloxacin, exposure in 12–18-year-olds is also relatively low as most children in that age group (i.e. 83–100%, data not shown) receive the maximum dose based on their weight, resulting in a lower mean mg/kg dose. In neonates, predicted drug exposure increases markedly with decreasing age and is thereby higher than in children, unless the dosage is reduced as, for example, with AMX IV (Table 1, Figure 2a). Neonatal dose reductions vary considerably (i.e. 0%–63%), so the effect on exposure relative to paediatric exposure varies similarly.

### Model predictions; PTA

**Figure 3.**
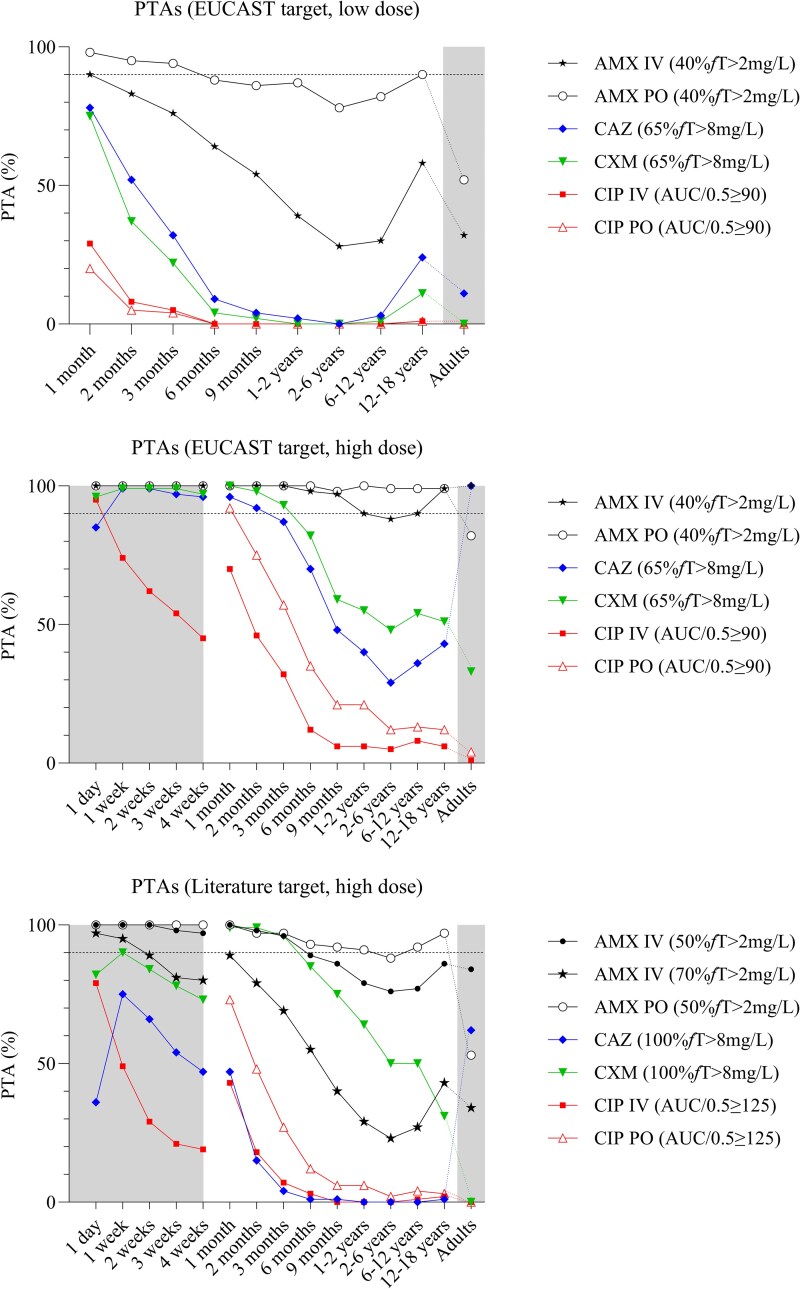
PTA on neonatal, paediatric and adult dosages. Simulated doses are provided in Table 1. The shaded area on the left indicates neonatal doses (b and c), and on the right, the adult doses (a–c). The dashed line represents a PTA of 90%. The applied PD targets are shown in the legends. For CXM, the task force high dose is used in (b), the DPF dose in (c). Abbreviations: AMX, amoxicillin; CAZ, ceftazidime; CIP, ciprofloxacin; CXM, cefuroxime; DPF, Dutch Paediatric Formulary; IV, intravenous; PO, oral.

The PTAs of all paediatric and adult dosages (based on EUCAST cut-off values and cut-off values reported in literature) are shown in Figure 3. The figure clearly illustrates that, based on simulations of worst-case scenarios, the highest antibiotic doses do not always achieve PD targets in >90% of simulated subjects. Not surprisingly, the PTAs for the low paediatric doses or of the more stringent therapeutic cut-off values reported in literature (as applied in critically ill patients) perform even worse (Figure 3a and c). In addition, the effect of age is significant, as simulations show that PTA decreases with age, reaching its lowest point between 2 and 6 years.

## Discussion

In response to the question of how paediatric dosages compare with adult EUCAST dosages, we showed with PBPK modelling and simulation that drug exposure in children is generally higher than in adults and that it is quite variable with age. For both low and high doses, exposure in older children is only slightly higher than in adults, but with decreasing age, exposure increases and the difference with adults enlarges. This indicates that the currently recommended paediatric dosages do not need to be increased.

In itself, this is not a surprising finding. When you convert the adult dose into a mg/kg dose; 6 grams of ceftazidime per day in a 70 kg adult is similar to 86 mg/kg, which is only 57% of the paediatric high dose of 150 mg/kg; a calculation that can also be applied to other antibiotics to get a quick impression of how adult and paediatric dosages roughly compare. In any case, paediatric dosages do not need to be increased based on exposure, but the question of whether such a higher exposure in children can be justified is very complex.

Further simulations exploring the PTAs of paediatric and adult doses of all drugs showed that infections caused by micro-organisms with an MIC at the breakpoint cannot all be effectively treated with current doses. And although a higher dose obviously results in a higher PTA, it should be questioned whether it is necessary to aim for a PTA of >90% in these cases before increasing dose. The PTA highly depends on two variables, i.e. the MIC and the PD target, making interpretation challenging.

The accuracy of MIC determinations is low due to technical assay variation and biological variation.^41,42^ MIC values follow a lognormal distribution, which should be taken into account when interpreting MICs. For most bacteria, the MIC can vary three to five 2-folds, meaning that for a measured MIC of 1 mg/L, the ‘true’ MIC could range from 0.5–2 mg/L (three 2-folds) or 0.25–4 mg/L (five 2-folds).^41,42^ Hence, it is generally recommended to treat an infection as if the MIC is as high as the breakpoint, which is why we used this value for our assessments, although realizing that only a minority of micro-organisms actually has such a high MIC.^40,41,43^

For the other variable, the PD target, consensus is lacking on the exact cut-off values. These cut-off values are mainly determined in preclinical dose-finding studies and translation of these studies into clinical practice is difficult. Thereby, cut-off values for PD targets seem to be influenced by the severity and location of the infection.^44^ However, regardless of the exact value of the PD target and associated PTAs, this study clarifies that current doses do not achieve consistent results during childhood. Just because we started from breakpoint, the effect of growth and development on achieving the PD target becomes clear. In general, the PTA in a 2-month-old infant is significantly higher than the PTA in a 2-year-old. At lower PD cut-off values (or lower MICs), PTAs logically increase markedly, but the 2-month-old infant will reach supratherapeutic levels with lower mg/kg doses than the 1-year-old infant, which is not visible because the PTA has then reached the maximum of 100% for both ages. This example also directly illustrates why PBPK modelling and simulating is ideally suited by including paediatric growth and maturation in assessing the PTA. In the future, when certainty exists on the measurement of MICs and the cut-off values for PD targets, PBPK simulations are a powerful tool to determine the correct, model-informed, dosage.

In addition to PBPK models, other PK models can be used to predict the efficacy of antibiotics in the paediatric population, with population PK (popPK) being the most commonly used. In contrast to PBPK modelling, popPK modelling is a top-down approach that relies on clinical PK data to which a model is fitted. To gain trust in our pragmatic PBPK approach, we also compared our outcomes with published popPK data and simulations. PBPK simulations indeed appeared equally suitable to inform dosing, as popPK studies yielded similar pharmacokinetic and PTA results as our data, and if results differed, this could be easily explained. For oral AMX, for example, popPK studies showed similar PTAs for comparable daily doses and PD targets.^23,24,45^ However, for IV AMX, popPK studies were quite heterogeneous in terms of dosing and PD cut-off values, which complicated comparison. Nevertheless, the results of Keij *et al.* differ significantly from those of van Donge *et al.*^23,46^ The model of Keij *et al.* predicted that administering 25 mg/kg two or three times daily results in a PTA of >90% in neonates 0–7 days and 7–27 days old, respectively, at a target of 50%T > 8 mg/L. By contrast, van Donge *et al.* showed that 25 mg/kg four times daily results in a PTA of ∼80% at that same target. This discrepancy is probably caused by the much lower elimination constant of Keij *et al.* (i.e. 0.075 h^−1^ compared with 0.21 h^−1^ of van Donge *et al.*). Van Donge *et al.* simulated multiple scenarios, showing similar results compared with our neonatal simulations, which were also consistent with the modelling results of Tang *et al.*^18^ Three popPK studies in children beyond the neonatal age also showed considerable variability. De Cock *et al.* and Lonsdale *et al.* studied a population with a broad age range, determining a single PTA across the age range from 1 month to adults.^19,47^ However, our simulations showed that age has a considerable impact on PTA due to maturation processes, indicating that certain age groups may either be under- or overdosed.

For CAZ, fewer popPK studies have been performed. Two neonatal popPK studies showed that 50 mg/kg three times daily is sufficient to achieve a PTA of >90% when aiming for a PD target of 100%*f*T > 8 mg/L.^26,48^ Van der Veer *et al.* specifically examined neonates undergoing controlled therapeutic hypothermia, which decreases CAZ clearance, possibly explaining the PTA seen at lower doses.^26^ By contrast, Leegwater *et al.* found that the 150 mg/kg/day dose would result in a PTA of 57% and that CAZ exposure was only sufficient when administered as continuous infusion, which is in line with our model predictions and those by Zhou *et al*.^49,50^ PopPK studies in older children used a lower PD target (50%*f*T > MIC), making direct comparison challenging.^51–53^ These studies found that the 150 mg/kg/day dose is sufficient to achieve adequate PTAs aimed at 50%*f*T > 8 mg/L, implying that this dose is not sufficient when aiming for a higher target.

For CXM IV only a few modelling studies have been performed in paediatrics, all involving children on cardiopulmonary bypass (CPB).^32,54,55^ Prophylactic CXM dosing during CPB, which is influenced by factors such as priming fluid volume and device settings, cannot be directly compared with therapeutic dosing, which is the focus of our study. Several PK simulation studies in adults, however, also found low PTAs at traditional dosing (i.e. 1500 mg every 8 hours, with an infusion duration of 15–30 minutes) and suggested increasing the dosing frequency, extending the infusion duration, or administering CXM as a continuous infusion, which aligns with our modelling results.^56–59^

The low PTAs predicted for CIP by our model also correspond to those found in other PK simulation studies.^33,34,39,60–62^ Oral doses up to 60 mg/kg/day were not sufficient to reach the therapeutic target.^33,34,39,61,62^ For the IV administration, simulation results varied: Meesters *et al.* predicted a 93% PTA for a 30 mg/kg/day dose, whereas Orito *et al.* predicted only 12% PTA with the same dose.^34,39^ These discrepancies are most likely partly caused by the much lower clearance (0.23 L/h/kg) and volume of distribution (0.55 L/kg) reported by Meesters *et al.* compared with others (0.43–1.46 L/h/kg and 2.09–9.89 L/kg, respectively).^33,34,39,60–65^ In addition, the renal contribution to total clearance in the model of Meesters *et al.* is only 50%, which is lower than the expected 67%.^34^ According to simulations of Facchin *et al.* and Hirt *et al.*, IV doses of 45–90 mg/kg/day would be required to achieve the therapeutic target, endorsing the findings of our study.^60,61^

Interestingly, from a clinical perspective, there are no signs that current dosages as recommended by EUCAST and DPF are inadequate. Clinical practice does suggest that in category ‘I’ infections, the lower dosage for category ‘S’ infections is not sufficient, supporting the EUCAST’s newly defined susceptibility categories and the adequacy of the paediatric high dosages as, for example, currently recommended in the DPF. Moreover, MICs are not always determined in practice, only the micro-organism and its susceptibility to selected antibiotics is tested. In the case of more severe infections and failing therapy, determination of the MIC for a specific antibiotic and therapeutic drug monitoring are important considerations.^66^ In those cases where EUCAST or high paediatric dosages do not result in clinical cure, one should also realize that not only less susceptible micro-organisms need higher dosages for eradication. The disease itself and/or certain treatment conditions, such as critical illness or CPB and hypothermia respectively, may alter drug PK, requiring dose adjustments to achieve clinical cure.^67^

In conclusion, current paediatric high dosages as recommended, among others, by the Dutch national paediatric dosing guideline (DPF) for the treatment of category ‘I’ infections are in line with adult dosage recommendations. PBPK model simulations indicated that antibiotic exposure in children is higher than in adults using currently used paediatric doses, reinforcing the basis for current dosing recommendations. In addition, model simulations showed that PTA varies by drug-micro-organism combination and age, and that children (especially those aged 2–6 years) may experience therapy failure for some of the tested drug–micro-organism combinations with MICs at the breakpoint. Clearly, our data indicate that in case of therapy failure or toxicity, individualization of doses should be considered in both adults and children, especially in severe infections with micro-organisms with known relatively high MICs. Therapeutic drug monitoring might be helpful here, although optimization of therapeutic cut-off values and more precise measurements of MICs may be prerequisites for real improvements in this area.

## Supplementary Material

dkaf463_Supplementary_Data

## Supplementary data

Figures S1 to S4 and Tables S1 to S6 are available as Supplementary data at *JAC* Online.
