# Correction to: Do the redefined EUCAST susceptibility categories warrant adjustment of paediatric antibiotic dosages? Pragmatic physiologically based pharmacokinetic modelling of four commonly used agents

**DOI:** 10.1093/jac/dkag249

**Published:** 2026-07-17

This is a correction to: Marika A de Hoop-Sommen, Jolien J M Freriksen, Joyce E M van der Heijden, Jens Jacobs, Yvette Oosterlaan, Chantal M Staring, Shannon L van der Zeeuw, Tjitske M van der Zanden, Jan-Tom van der Bruggen, Marjolijn S W Quaak, Clementien Vermont, Tom F W Wolfs, Roger J M Brüggemann, Rick Greupink, Saskia N de Wildt, Do the redefined EUCAST susceptibility categories warrant adjustment of paediatric antibiotic dosages? Pragmatic physiologically based pharmacokinetic modelling of four commonly used agents, *Journal of Antimicrobial Chemotherapy*, Volume 81, Issue 2, February 2026, dkaf463, https://doi.org/10.1093/jac/dkaf463

In the originally published version of this manuscript, the route of administration for cefuroxime was incorrectly reported in Table 1.

This has been corrected to read “IV” instead of “PO”.

