# Supplementary material for: Do the redefined EUCAST susceptibility categories warrant adjustment of paediatric antibiotic dosages? Pragmatic physiologically based pharmacokinetic modelling of four commonly used agents
Source: J Antimicrob Chemother. 2026 Jan 29;81(2):dkaf463. doi: 10.1093/jac/dkaf463 (PMC12853878; doi:10.1093/jac/dkaf463)
Supplement: dkaf463_Supplementary_Data [file dkaf463_supplementary_data.docx]

Supplementary material

Do the redefined EUCAST susceptibility categories warrant adjustment of paediatric antibiotic dosages? Pragmatic physiologically-based pharmacokinetic modelling of four commonly used agents

Authors: Marika A. de Hoop-Sommen^1^, Jolien J.M. Freriksen^1^, Joyce E.M. van der Heijden^1^, Jens Jacobs^1^, Yvette Oosterlaan^1^, Chantal Staring^1^, Shannon van der Zeeuw^1,2^, Tjitske M. van der Zanden^1,3,4^, Tjomme. van der Bruggen^5^, Marjolijn S.W. Quaak^6,7^, Clementien Vermont^7^, Tom F.W. Wolfs^8^, Roger J.M. Brüggemann^1,9^, Rick Greupink^1^, Saskia N. de Wildt*^1,3,10^

^1^Department of Pharmacy, Radboud university medical centre, Nijmegen, The Netherlands.

^2^Department of Hospital Pharmacy, Erasmus MC, Rotterdam, The Netherlands.

^3^Department of Paediatric and Neonatal Intensive Care, Erasmus MC-Sophia Children’s Hospital, Rotterdam, The Netherlands.

^4^Dutch Knowledge Centre Pharmacotherapy for Children, The Hague, The Netherlands.

^5^Department of Medical Microbiology, University Medical Centre Utrecht, Utrecht, The Netherlands.

^6^Department of Paediatrics, Dijklander Ziekenhuis, Hoorn, The Netherlands

^7^Division of Infectious Diseases and Immunology, Department of Paediatrics, Erasmus MC University Medical Centre-Sophia Children's Hospital, Rotterdam, The Netherlands.

^8^Department of Paediatrics, Wilhelmina Children's Hospital, Utrecht, the Netherlands.

^9^Radboud Institute for Medical Innovation, Radboud university medical centre, Nijmegen, The Netherlands.

^10^Department of Intensive Care, Radboud university medical centre, Nijmegen, The Netherlands.

*Correspondence: [saskia.dewildt@radboudumc.nl](mailto:saskia.dewildt@radboudumc.nl)

Content

[1. Model input parameters 2](#_Toc194595851)

[2. Literature search queries 6](#_Toc194595852)

[3. Pharmacokinetic studies 7](#_Toc194595853)

[4. Model verification 12](#_Toc194595854)

[Amoxicillin 13](#_Toc194595855)

[Cefuroxime 18](#_Toc194595856)

[Ciprofloxacin 21](#_Toc194595857)

[5. References 29](#_Toc194595858)

# 1. Model input parameters

Table S1. PBPK model input parameters

|  | **Parameter** | **Amoxicillin ^1^** | **Ceftazidime ^3^** | **Cefuroxime ^1^** | **Ciprofloxacin ^2^** |
| --- | --- | --- | --- | --- | --- |
| Phys. properties | Molecular weight (g/mol) | 365.1 | 546.6 | 424.39 | 331.4 |
|  | logP | 0.87 | -3.75 | -0.90 | 0.3 |
|  | Compound type | Ampholyte | Diprotic acid | Monoprotic acid | Ampholyte |
|  | pKa 1, pKa 2 | 3.23, 7.43 | 2.43, 2.89 | 3.15, NA | 6.09, 8.74 |
|  | B/P | 0.55 (UI) | 0.55 (UI) | 0.56 (UI) | 0.75 (UI) |
|  | Fraction unbound in plasma, fu | 0.75 (P) | 0.85 (UI) | 0.67 (UI) | 0.786 (UI) |
|  | Main plasma binding protein | HSA | HSA | HSA | HSA |
| Absorption | Absorption model | First-order | - | - | ADAM |
|  | Fraction absorbed, fa | 0.55 (30% CV) (UI) |  |  | 0.99 |
|  | Absorption rate constant, ka (1/h) | 0.6 (30% CV) (UI) |  |  | 1.89 |
|  | Lag time (h) | 0.5 (UI) |  |  | 0 |
|  | fu_gut_ | 1 (UI) |  |  | 0.786 (UI) |
|  | Q_gut_ (L/h) | 0.65 (P) |  |  | 12.666 (P) |
|  | P_eff,man_ (10^-4^cm/s) | 0.076 (P) |  |  | 4.33 (UI) |
|  | Topological Polar Surface Area (Å²) | 133 |  |  | 72.88 |
|  | Hydrogen Bond Donor Count | 4 |  |  | 2 |
| Distribution | Distribution model | Full PBPK model | Full PBPK model | Full PBPK model | Full PBPK model |
|  | Vss (L/kg) | 0.189 (P) | 0.195 (P) | 0.17 (P) (adults)  0.68 (P) (paediatrics) | 2.35 (P) |
|  | Prediction method | Method 2 | Method 2 | Method 2 | Method 2 |
|  | Kp scalar | 0.55 | 1 | 0.7 (adults)  3.495 (paediatrics) | 6.33 |

Table S1 continued.

|  | **Parameter** | **Amoxicillin ^1^** | **Ceftazidime ^3^** | **Cefuroxime ^1^** | **Ciprofloxacin ^2^** |
| --- | --- | --- | --- | --- | --- |

| Elimination | Metabolism model | Enzyme kinetics | Enzyme kinetics | Enzyme kinetics | Enzyme kinetics |
| --- | --- | --- | --- | --- | --- |
|  | Enzyme (CL_int_ (µL/min/pmol)) | - | - | - | CYP1A2 (0.1369) |
|  | CL_int_ (Bile) (µL/min/10^6^cells) | 0.1 | 0 | 0 | 0.651 |
|  | Renal elimination model | Mech KiM |  | Mech KiM off | Mech KiM |
|  | fu Kidney Cell | 1 (P) |  | 1 (P) | 1 (P) |
|  | fu Urine | 1 (default) |  | 1 (default) | 1 (default) |
|  | Kidney Transporter OAT1 (SLC22A6) |  |  | Basolateral (uptake)  CL_int,T_: 9.62 µL/min/10^6^cells  User (RAF/REF=1) |  |
|  | Kidney Transporter OAT3 (SLC22A8) | Basolateral (uptake)  CL_int,T_: 14 µL/min/10^6^cells  User (RAF/REF=1) |  |  | Basolateral (uptake)  CL_int,T_: 35 µL/min/10^6^cells  User (RAF/REF=1) |
|  | Kidney Transporter MRP4 (ABCC4) |  |  | Apical (Efflux)  CL_int,T_: 10 µL/min/10^6^cells  User (RAF/REF=1) |  |
|  | CL_PD,uu,basal & apical_ (mL/min/10^6^ PT cells) | 6.44E-06 |  | 0 |  |
|  | CL_R_ (L/h) | 11.25 | 6 (UI) | 11 |  |
|  | Additional systemic CL (L/h) | 0 | 0.9 (UI) | 0 |  |

Abbreviations: ADAM: Advanced Dissolution, Absorption and Metabolism, B/P: blood-to-plasma partition ratio, CL_int_: *in vitro* intrinsic clearance, CL_PD basal_: passive diffusion clearance, CL_R_: renal clearance, fu_gut_: unbound fraction of drug in enterocytes, HSA: human serum albumin, Kp: tissue-plasma partition coefficients, Mech KiM: Permeability-limited kidney model, MRP: multidrug resistance-associated protein, NA: not applicable, OAT: organic anion transporter, P: predicted, P_eff,man_: human jejunum effective permeability, pKa: acid dissociation constant, PT: proximal tubular, Q_gut_: nominal flow in gut model, UI: user input, Vss: volume of distribution at steady state using tissue volumes for a population representative of healthy volunteers population

Adjustment from original model in red

The ontogeny profiles for CYP1A2, OAT1, OAT3 and MRP4 were defined as depicted in Figure S1.


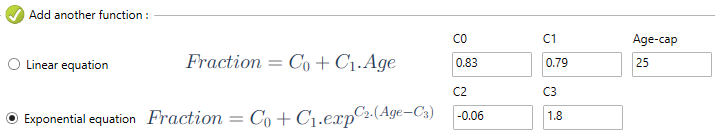

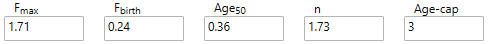

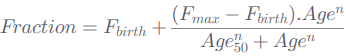

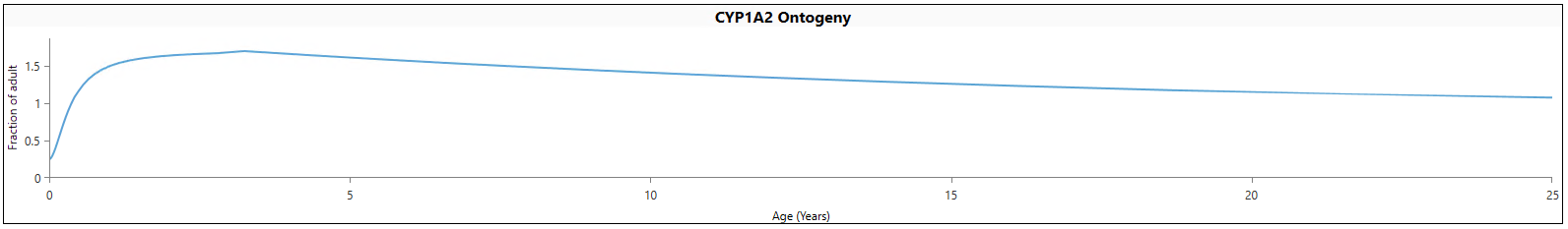


A

B


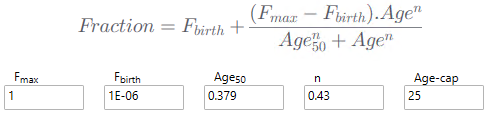

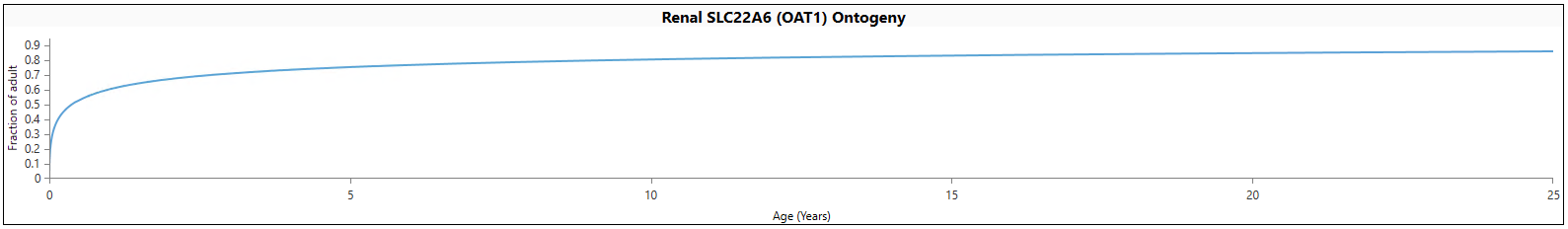


C


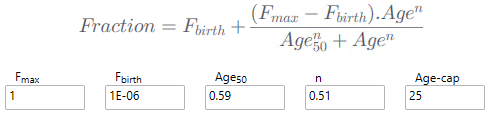

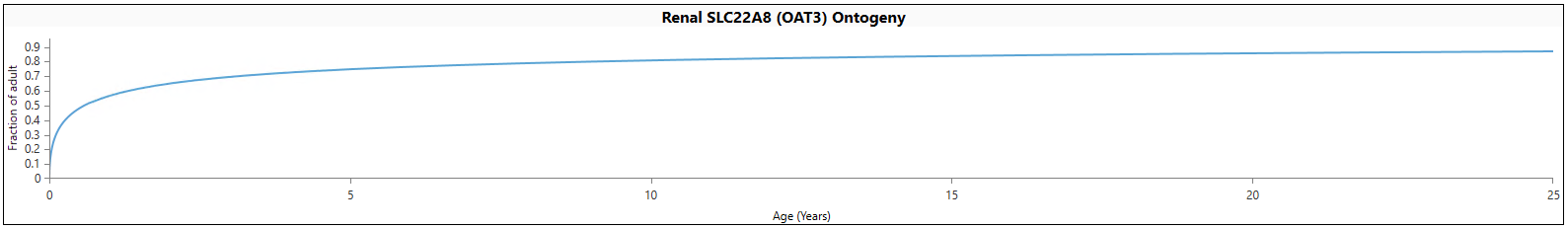


**Figure S1.** Ontogeny profiles of enzymes and transporters. Simcyp’s default ontogeny profiles were used for CYP1A2 (A), OAT1 (B), OAT3 (C), and MRP4 (D)


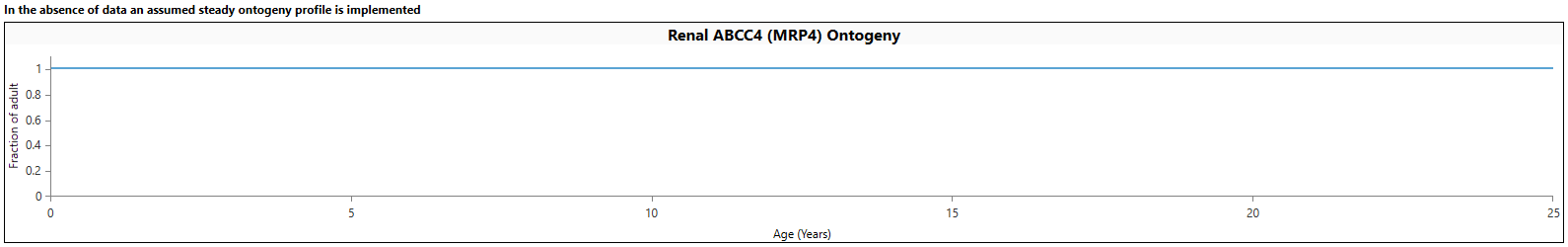


D

**Figure S1 continued**. Ontogeny profiles of enzymes and transporters. Simcyp’s default ontogeny profiles were used for CYP1A2 (A), OAT1 (B), OAT3 (C), and MRP4 (D)

# 2. Literature search queries

Two standardized PubMed search queries were designed to find PK data of each drug; one for adults and one for paediatrics. Similar articles and references were checked for any other relevant publications that were not found with the initial search queries.

Adults:

((Pharmacokinet*[Title/Abstract]) AND (DRUGNAME*[Title] OR BRANDNAME*[Title]) AND (Healthy[Title] OR volunteer*[Title] OR adult*[Title] OR subject*[Title] OR Man[Title] OR Men[Title] OR Woman[Title] OR Women[Title] OR male*[Title] OR female*[Title])) OR (("Pharmacokinetics of"[Title]) AND ("DRUGNAME"[Title] OR "BRANDNAME"[Title]) OR (“DRUGNAME pharmacokinetics”[Title]))

Paediatrics:

(Pharmacokinet*[Title/Abstract]) AND (DRUGNAME*[Title] OR BRANDNAME*[Title]) AND (Infan*[Title] OR newborn*[Title] OR new-born*[Title] OR perinat*[Title] OR neonat*[Title] OR baby[Title] OR baby*[Title] OR babies[Title] OR prematur*[Title] OR preterm*[Title] OR toddler*[Title] OR minors[Title] OR minors*[Title] OR boy[Title] OR boys[Title] OR boyfriend[Title] OR boyhood[Title] OR girl*[Title] OR kid[Title] OR kids[Title] OR child[Title] OR child*[Title] OR children*[Title] OR schoolchild*[Title] OR schoolchild[Title] OR school child[Title] OR school child*[Title] OR adolescen*[Title] OR juvenil*[Title] OR youth*[Title] OR teen*[Title] OR under*age*[Title] OR pubescen*[Title] OR pediatrics[MeSH] OR pediatric*[Title] OR paediatric*[Title] OR peadiatric*[Title])

Table S1 shows for each drug the drug and brand names, the date of the search, and the number of search results.

###### Table S2. Search results

| Drug name | Brand name | Date of search | Search results adults | Search results paediatrics |
| --- | --- | --- | --- | --- |
| Amoxicillin | Augmentin | 2023-05-15 | 114 | 52 |
| Ceftazidime | Fortum | 2021-08-25 | 117 | 43 |
| Cefuroxime | Zinnat | 2023-01-31 | 68 | 10 |
| Ciprofloxacin | Ciproxin | 2022-12- | 257 | 29 |

# 3. Pharmacokinetic studies

###### Table S3a. Pharmacokinetic studies used for model verification of amoxicillin in adults

| **Study** | **Design** | **N** | **Dose** | **Administration** | **Health**  **status** | **Age range**  **(years)** | **Comed.** | **Fasted/**  **fed** | **Prop. of females** | **Ref** |
| --- | --- | --- | --- | --- | --- | --- | --- | --- | --- | --- |
| **IV administration adults** | | | | | | | | | | |
| Arancibia 1980 | SD | 9 | 500 mg | IV bolus 10 sec | Healthy | 21-45 | None | *na* | 0.22 | ^5^ |
| Paintaud 1992 | SD | 6 | 500 mg | IV bolus 5 min | Healthy | *ns* | None | *na* | 0.33 | ^6^ |
| Witkowski 1982 | SD | 10 | 500 mg | IV bolus, time *ns* | Healthy | 25-44 | *ns* | *na* | 0.5 | ^7^ |
| Zarowny 1974 | SD | 8 | 250 mg | IV infusion 33 min | Healthy | 20-30 | *ns* | *na* | 0 | ^8^ |
| **PO administration adults** | | | | | | | | | | |
| Arancibia 1980* | SD | 9 | 500 mg | Capsule | Healthy | 21-45 | None | Fasted | 0.22 | ^5^ |
| Ghim 2021 | MD | 19 | 1000 mg q12h | Capsule | Healthy | 19-45 | *ns* | Fasted | 0 | ^9^ |
| Liew 2014* | SD | 18 | 500 mg | Capsule | Healthy | 24-38 | *ns* | Fasted | 0 | ^10^ |
| Padoin 1995 | SD | 12 | 1000 mg | Capsule | Healthy | 19-39 | *ns* | Fasted | 0 | ^11^ |
| Paintaud 1992* | SD | 6 | 500 & 3000 mg | Suspension | Healthy | *ns* | None | Fasted | 0.33 | ^6^ |
| Perveen 2022 | SD | 12 | 250 mg | Capsule | Healthy | 21-28 | *ns* | Fasted | 0 | ^12^ |
| Ullah 2008* | SD | 24 | 500 mg | Capsule | Healthy | 23-34 | *ns* | Fasted | 0 | ^13^ |
| Witkowski 1982* | SD | 10 | 500 mg | Capsule | Healthy | 25-44 | *ns* | Fasted | 0.5 | ^7^ |
| Zarowny 1974 | SD | 8 | 250 mg | Capsule | Healthy | 20-30 | *ns* | Fasted | 0 | ^8^ |

* Pooled simulation of 500 mg SD PO fasted in 21-45 y, 50% females. Abbreviations: IV: intravenous, MD: multi dose, *na*: not applicable, *ns*: not specified, PO: oral, q12h: every 12 hours, SD: single dose

###### Table S3b. Pharmacokinetic studies used for model verification of amoxicillin in paediatrics

| **Study** | **Design** | **N** | **Dose** | **Administration** | **Health status** | **Age range** | **Fasted/ Fed** | **Prop. of**  **females** | **Ref** |
| --- | --- | --- | --- | --- | --- | --- | --- | --- | --- |
| **IV administration** | | |  |  |  |  |  |  |  |
| Schaad 1983 | SD | 12 | 25 mg/kg | Bolus 2 min | Viral infection, neurological diseases | 2-14.5 y | *na* | 0.42 | ^14^ |
| Vargas 2004 | SD | 11 | 30 mg/kg | Bolus, time *ns* | Cardiopulmonary bypass surgery (hypothermia) | 3-60 d | *na* | *ns* | ^15^ |
| Rudoy 1979 | MD | 14 | 14.8 mg/kg q6h  26.4 mg/kg q6h  41.5 mg/kg q6h | Infusion 20-30 min | Proven/suspected infections | Mean 16 mo  Mean 10 mo  Mean 36 mo | *na* | *ns* | ^16^ |
| **PO administration** | | |  |  |  |  |  |  |  |
| Fonseca 2003 | SD+MD | 66 | 15 mg/kg q8h  25 mg/kg q12h | Suspension | Non-severe pneumonia | 5-52 mo  3-48 mo | 1 h fasted | 0.41  0.5 | ^17^ |
| Ginsburg 1979 | MD | 24 | 15 mg/kg q6h  25 mg/kg q8h | Suspension | OMA | 4-45 mo | Fasted and fed | *ns* | ^18^ |
| Marks 1978 | SD | 10  10 | 12.5 mg/kg  25 mg/kg | Suspension | Recurrent UTI/OMA | 3.5-12 y | Various | ‘At least 20 girls’ 🡪 0.54 | ^19^ |
| Nelson 1982 | SD | 34 | 6.6 mg/kg  13.3 mg/kg | Suspension | OMA or skin infections | *ns* 🡪 1 mo – 11y* | *ns* | *ns* | ^20^ |
| Van Niekerk 1985 | SD | 35 | 125 mg  250 mg | Suspension | Non-severe infections | 2.08-5.92 y  6.25-9.5 y | *ns* | 0.56  0.58 | ^21^ |
| Schaad 1986 | SD | 11 | 20 mg/kg | Syrup | Various non-bacterial diseases | 3.1-13.8 y | Fasted | 0.64 | ^22^ |

*Assumption, as Nelson et al. only specified “infants and children”. Abbreviations: d: days, IV: intravenous, mo: months, *na*: not applicable, *ns*: not specified, OMA: otitis media acuta, PO: oral, qXh: every x hours, UTI: urinary tract infection, y: year

###### Table S4a. Pharmacokinetic studies used for model verification of cefuroxime in adults

| **Study** | **Design** | **N** | **Dose** | **Administration** | **Health status** | **Age range**  **(years)** | **Comed.** | **Fasted /fed** | **Prop. of females** | **Ref** |
| --- | --- | --- | --- | --- | --- | --- | --- | --- | --- | --- |
| **IV administration** | |  |  |  |  |  |  |  |  |  |
| Bundtzen 1981 | SD | 12 | 750 mg | IV bolus 2 min | Non-severely ill | 46-91 | *ns* | *na* | 0 | ^23^ |
| Carlier 2014 | MD | 20 | 1500 mg q8h | IV infusion 30 min | Critically ill | 26-85 | *ns* | *na* | 0.27 | ^24^ |
| Gower 1977 | SD | 6 | 500 mg  750 mg | IV bolus 2-3 min | Healthy | 28-38 | None | *na* | 0 | ^25^ |
| Hosmann 2018 | MD | 6 | 1500 mg q8h | IV infusion 60 min | Prophylaxis CNS infection | 52.7 ± 14.3 | *ns* | *na* | 0.83 | ^26^ |
| Nascimento 2007 | MD | 17 | 1500 mg q12h | IV bolus, time *ns* | Cardiac surgery | 50.7 ± 9.1 | *ns* | *na* | 0.35 | ^27^ |
| Schwameis 2017 | SD | 10 | 1500 mg | IV infusion 30 min | Elective knee arthroscopy | 20-61 | *ns* | *na* | 0.2 | ^28^ |

Abbreviations: CNS: central nervous system, IV: intravenous, MD: multi dose, min: minutes, *na*: not applicable, *ns*: not specified, qXh: every x hours, SD: single dose

###### Table S4b. Pharmacokinetic studies used for model verification of cefuroxime in paediatrics

| **Study** | **Design** | **N** | **Dose** | **Administration** | **Health status** | **Age range** | **Comed.** | **Fasted**  **/fed** | **Prop. of**  **females** | **Notes** | **Ref** |
| --- | --- | --- | --- | --- | --- | --- | --- | --- | --- | --- | --- |
| **IV administration** | |  |  |  |  |  |  |  |  |  |  |
| Del Rio 1982 | SD  MD | 18  20  5 | 50 mg/kg  75 mg/kg  50 mg/kg q6h | IV infusion 15 min | Meningitis | 4 wk – 6.5 y | Standard therapy | *ns* | 0.32 |  | ^29^ |
| Knoderer 2011 | SD+MD | 15 | 25 mg/kg +  12 mg/kg once* | IV bolus, time *ns* | Cardiovascular disease | 3-33.6 mo | *ns* | *ns* | 0.4 | On CPB (hypothermia) | ^30^ |
| Nelson 1982 | MD | 87 | 25 mg/kg q8h | IV infusion 15 min | Pneumonia | 2 wk – 13.5 y | *ns* | *ns* | 0.45 |  | ^31^ |
| Olguín 2008 | SD | 5  6  4 | 25 mg/kg | IV infusion 30 min | Multiple organ system failure  Pharyngoamygdalitis | 4-23 mo  6-168 mo  5-11 y | *ns* | *ns* | 0.8  0.5  0.5 | Severely ill  Intubated  Control | ^32^ |
| Sorin 1977 | SD | 7 | 12 mg/kg | IV bolus 1-3 min | Severe infection | 3 wk – 24 mo | gentamicin (n=3) | *ns* | 0.48 |  | ^33^ |

*Second dose was administered in the cardiopulmonary bypass prime solution. Abbreviations: CPB: cardiopulmonary bypass, IV: intravenous, MD: multi dose, min: minutes, mo: months, *ns*: not specified, qXh: every x hours, SD: single dose, wk: weeks, y: year

###### Table S5a. Pharmacokinetic studies used for model verification of ciprofloxacin in adults

| **Study** | **Design** | **N** | **Dose** | **Administration** | **Health status** | **Age range (years)** | **Comed.** | **Fasted**  **/fed** | **Prop. of females** | **Ref** |
| --- | --- | --- | --- | --- | --- | --- | --- | --- | --- | --- |
| **IV administration** |  |  |  |  |  |  |  |  |  |  |
| Bergan 1986 | SD | 12 | 100 mg | IV bolus 3 min | Healthy | 21-40 | no | *na* | 0.5 | ^34^ |
| Borner 1986 | SD | 32 | 50 & 100 mg  200 mg | IV infusion 15 min  IV infusion 20 min | Healthy | 21-35 | *ns* | *na* | 0.5 | ^35^ |
| Gonzalez 1985 | MD | 9 | 100, 150 & 200 mg q12h | IV infusion 30 min | Healthy | 18-46 | *ns* | *na* | 0 | ^36^ |
| Höffken 1985 | SD | 12 | 50 & 100 mg | IV infusion 15 min | Healthy | 22-34 | *ns* | *na* | 0.5 | ^37^ |
| Lipman 1998 | SD+MD | 16 | 400 mg q8h | IV infusion 60 min | Severe sepsis | 18-54 | *yes, ns* | *na* | *ns* | ^38^ |
| Shah 1995 | SD+MD | 12 | 400 mg q8h | IV infusion 60 min | Healthy | 18-40 | *ns* | *na* | 0.5 | ^39^ |
| **PO administration** |  |  |  |  |  |  |  |  |  |  |
| Bergan 1986 | SD | 12 | 100, 250, 500 & 1000 mg | PO tablets | Healthy | 21-40 | none | Fasted | 0.5 | ^34^ |
| Borner 1986 | SD | 32 | 100, 250, 500 & 750 mg  250 mg | PO tablets | Healthy | 21-35 | *ns* | Fasted  Fed | 0.5 | ^35^ |
| Gonzalez 1984 | MD | 9 | 250, 500 & 750 mg q12h | PO tablets | Healthy | 19-45 | *ns* | 1^st^ dose: fasted  2^nd^ dose: fed | 0 | ^40^ |
| Höffken 1985 | SD | 12 | 50, 100 & 750 mg | PO tablets | Healthy | 22-34 | *ns* | Fasted | 0.5 | ^37^ |
| Israel 1993 | MD | 14 | 500 & 750 mg q12h | PO tablets | Healthy | 25 ± 3.6 | none | 1^st^ dose: fasted | 0 | ^41^ |
| LeBel 1986 | SD | 12 | 500 mg | PO tablets | Healthy | 19-25 | none | Fasted | 0.5 | ^42^ |

Abbreviations: IV: intravenous, MD: multi dose, min: minutes, *na*: not applicable, *ns*: not specified, PO: oral, qXh: every x hours, SD: single dose

###### Table S5b. Pharmacokinetic studies used for model verification of ciprofloxacin in paediatrics

| **Study** | **Design** | **N** | **Dose** | **Administration** | **Health status** | **Age range** | **Comed.** | **Fasted**  **/ Fed** | **Prop. of**  **females** | **Ref** |
| --- | --- | --- | --- | --- | --- | --- | --- | --- | --- | --- |
| **IV administration** | | |  |  |  |  |  |  |  |  |
| Lipman 2002 | MD | 10  10 | 10 mg/kg q12h | IV infusion 60 min | Severe sepsis | 3-12 mo  1.1-4.75 y | Metronidazole, vancomycin and anti-fungal agents permitted | *na* | 0.7  0.7 | ^43^ |
| Rubio 1997 | MD | 13  5 | 10 mg/kg q8h,  max 400 mg/dose | IV infusion 60 min | Pseudomonas exacerbation in CF | 5-12 y  13-17 y | No interacting drugs allowed | *na* | 0.39 | ^44^ |
| Schaefer 1996 | MD | 10 | 10 mg/kg q12h,  max 400 mg/dose | IV infusion 30 min | CF patients | 6-16 y | If needed, inhaled amikacin | *na* | *ns* | ^45^ |
| **PO administration** | |  |  |  |  |  |  |  |  |  |
| Peltola 1992 | SD | 7  9 | 15 mg/kg | PO ground tablet | Infection | 5-14 wk  1-5 y | *ns* | Food couple  hours later | 0.43  0.44 | ^46^ |
| Peltola 1998 | MD | 4  3  6  3 | 10 mg/kg q8h | PO suspension | Infection | 0.3-0.8 y  1.1-1.6 y  2.5-5.3 y  6.3-7.1 y | Kept at a minimum | ≥1 hour apart  from food | 0.5  0.33  0.67  1 | ^47^ |
| Rubio 1997 | MD | 13  5 | 20 mg/kg q12h,  max 1 g/dose | PO, formulation *ns* | Pseudomonas exacerbation in CF | 5-12 y  13-17 y | No interacting drugs allowed | *ns* | 0.39 | ^44^ |
| Schaefer 1996 | MD | 10 | 15 mg/kg q12h | PO, formulation *ns* | CF patients | 6-16 y | If needed, inhaled amikacin | Fasted | *ns* | ^45^ |

Abbreviations: CF: cystic fibrosis, IV: intravenous, MD: multi dose, min: minutes, mo: months, *na*: not applicable, *ns*: not specified, PO: oral, qXh: every x hours, SD: single dose, wk: weeks, y: year

#

# 4. Model verification

**Table S6a. Predicted-to-observed PK parameter ratios within 2, 1.5, and 1.25-fold.**

| With CPB | **AMX** | | | | **CAZ** | | | |
| --- | --- | --- | --- | --- | --- | --- | --- | --- |
|  | N | 2-fold | 1.5-fold | 1.25-fold | N | 2-fold | 1.5-fold | 1.25-fold |
| All | 147 | 90% | 54% | 35% | 72 | 96% | 75% | 56% |
| Adults | 50 | 90% | 68% | 46% | 45 | 100% | 93% | 89% |
| Children | 94 | 93% | 49% | 30% | 15 | 80% | 47% | 0% |
| Neonates | 3 | 33% | 0% | 0% | 12 | 100% | 42% | 25% |
|  | **CXM** | | | | **CIP** | | | |
|  | N | 2-fold | 1.5-fold | 1.25-fold | N | 2-fold | 1.5-fold | 1.25-fold |
| All | 97 | 80% | 66% | 54% | 223 | 97% | 81% | 54% |
| Adults | 30 | 93% | 80% | 73% | 138 | 99% | 83% | 62% |
| Children | 61 | 77% | 62% | 48% | 85 | 93% | 76% | 42% |
| Neonates | 6 | 50% | 33% | 17% | 0 | - | - | - |

Abbreviations: AMX: amoxicillin, CAZ: ceftazidime, CIP: ciprofloxacin, CPB: cardiopulmonary bypass, CXM: cefuroxime

**Table S6b.** **Predicted-to-observed PK parameter ratios within 2-fold for all four drugs.**

|  | **Totals** | |
| --- | --- | --- |
|  | N | 2-fold |
| Adults | 263 | 97% |
| Children | 255 | 88% |
| Neonates | 21 | 71% |

Abbreviation: CPB: cardiopulmonary bypass

## Amoxicillin


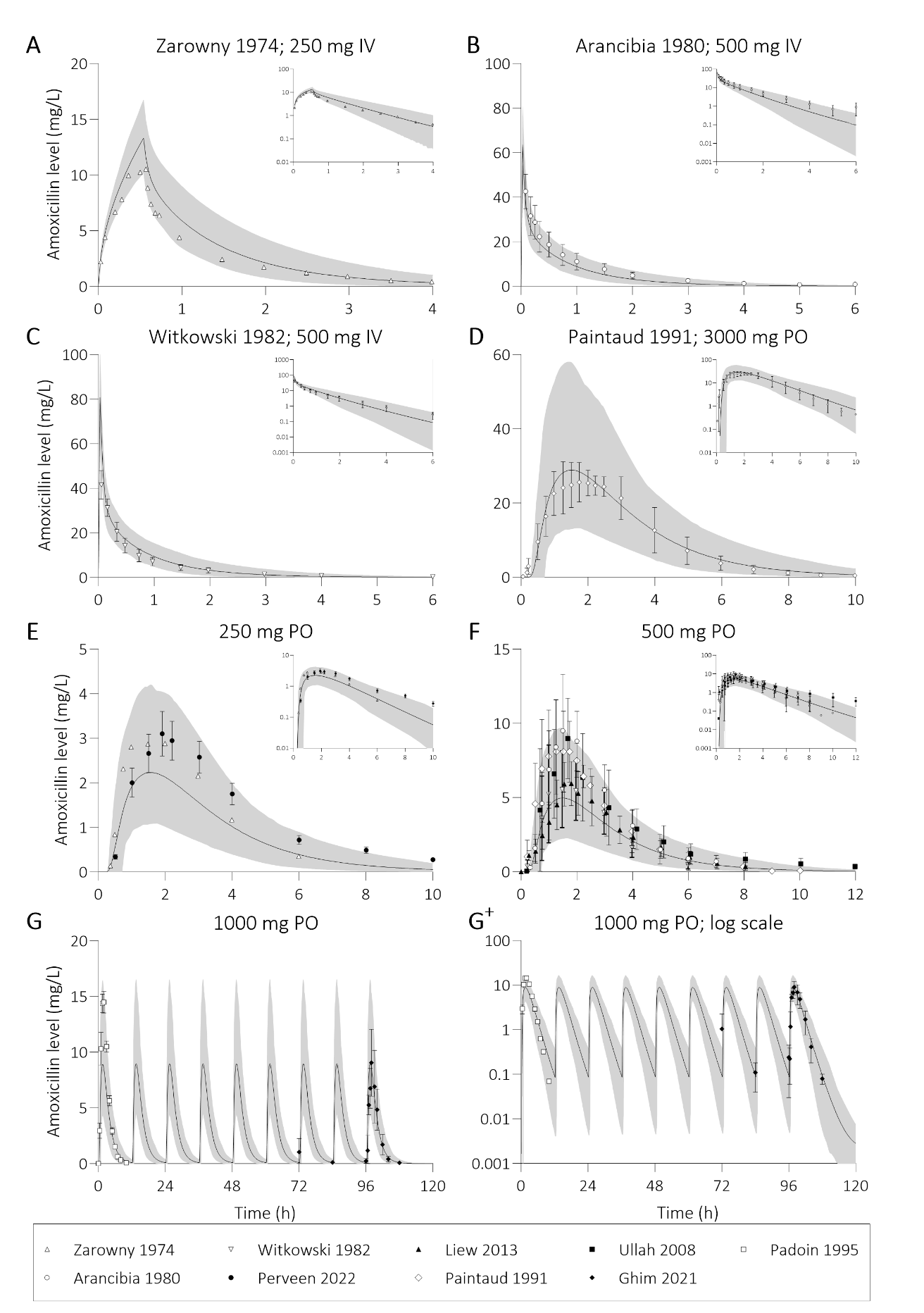


**Figure S2a. Visual predictive checks for amoxicillin in adults (single and multidose, IV and PO)**. The solid line is the predicted mean of the simulated population and the shaded area represents the 5^th^ to 95^th^ percentile of the virtual population. Symbols are mean observed datapoints ± standard deviation (Witkowski, Paintaud, Ullah, Ghim) or standard error of the mean (Arancibia, Padoin), inserts in A-F show semi-log plots, while G^+^ is the semi-log plot of G. ^5-13^ Abbreviations: IV: intravenous, PO: oral.


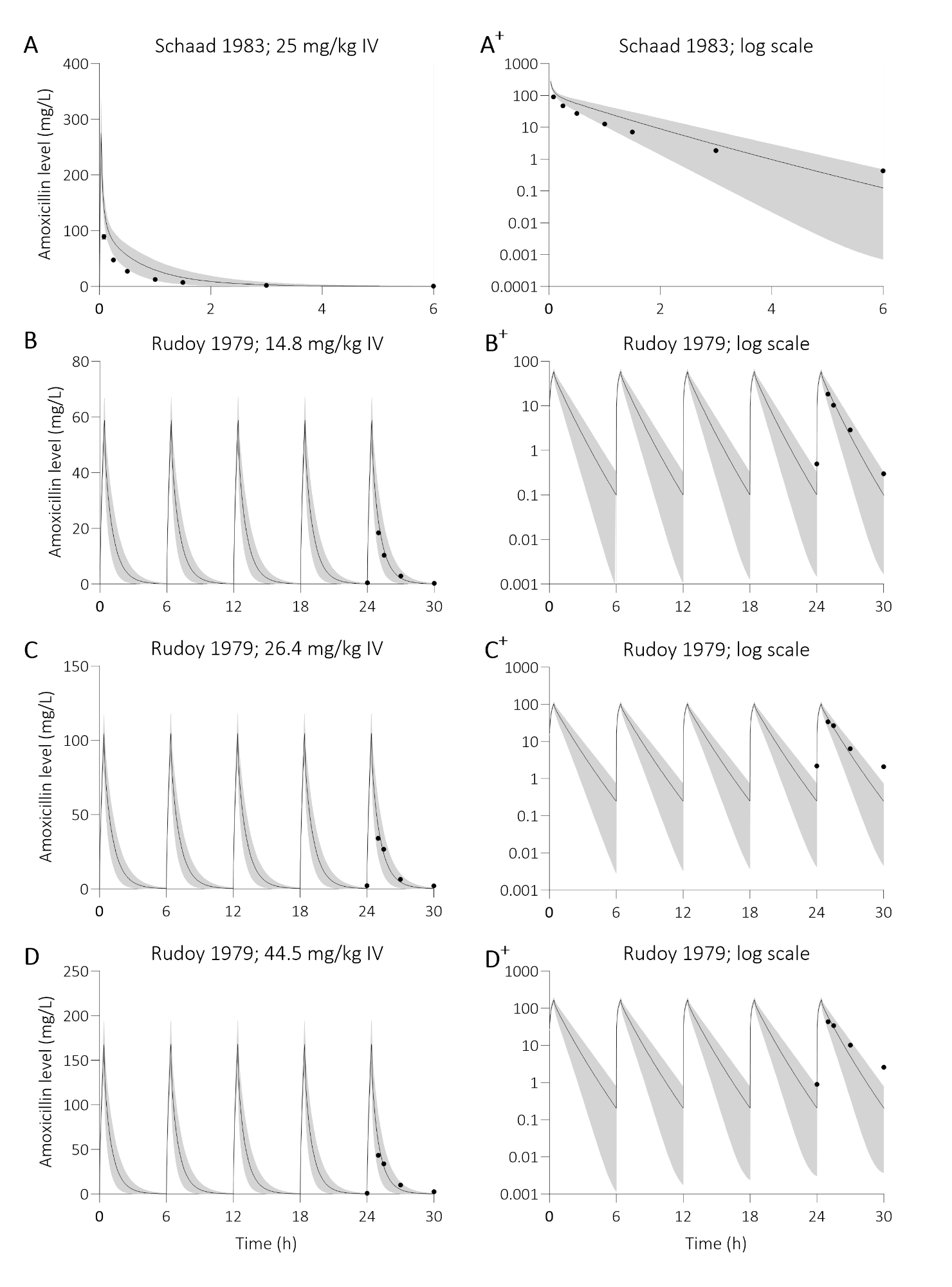


**Figure S2b. Visual predictive checks for amoxicillin in paediatrics (single and multidose IV).** The solid line is the predicted mean of the simulated population and the shaded area represents the 5^th^ to 95^th^ percentile of the virtual population. Symbols are mean observed datapoints. ^14, 16^ A^+^, B^+^, C^+^, and D^+^ are semi-log plots of A, B, C, and D, respectively. Abbreviation: IV: intravenous.


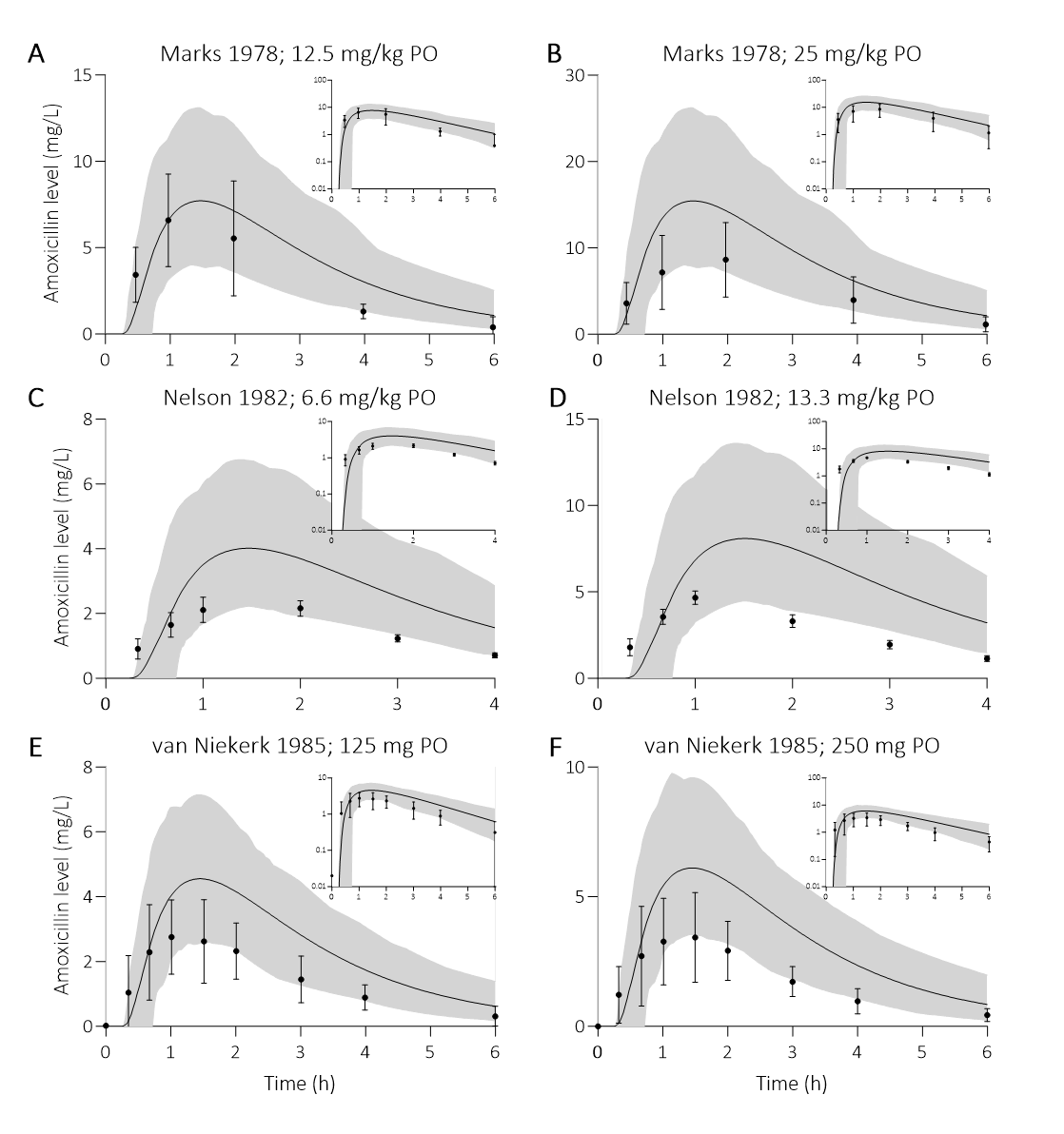


**Figure S2c.** **Visual predictive checks for amoxicillin in paediatrics (single dose PO).** The solid line is the predicted mean of the simulated population and the shaded area represents the 5^th^ to 95^th^ percentile of the virtual population. Symbols are mean observed datapoints ± standard deviation (A, B, E, F) or standard error of the mean (C, D), inserts show semi-log plots. ^19-21^ Abbreviation: PO: oral.


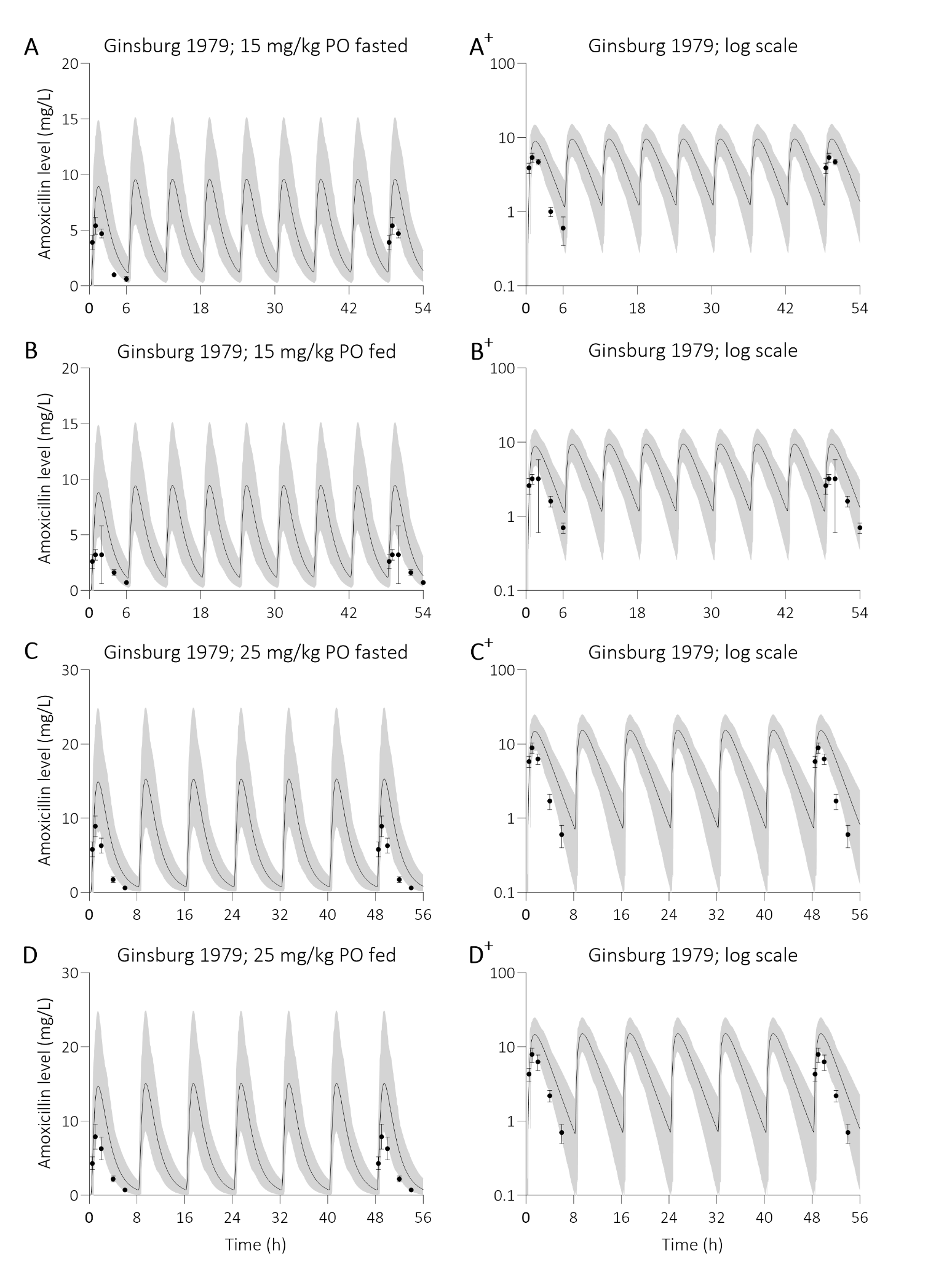


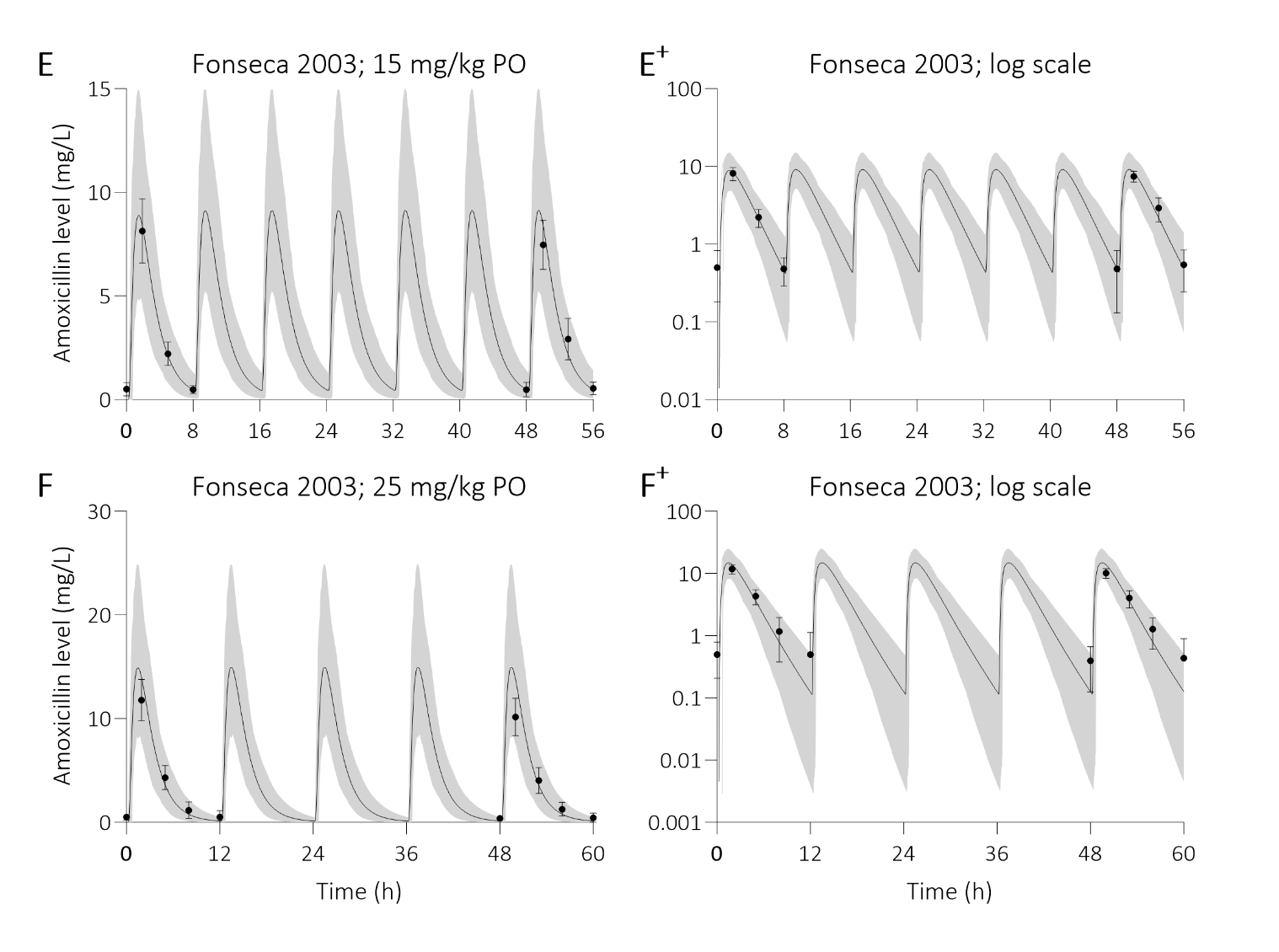


**Figure S2d.** **Visual predictive checks for amoxicillin in paediatrics (multidose PO).** The solid line is the predicted mean of the simulated population and the shaded area represents the 5^th^ to 95^th^ percentile of the virtual population. Symbols are mean observed datapoints + 95% confidence interval (E, E^+^, F, F^+^) or ± standard error. ^17, 18^ A^+^, B^+^, C^+^, D^+^, E^+^, and F^+^ are semi-log plots of A, B, C, D, E, and F, respectively. Abbreviation: PO: oral.

## Cefuroxime


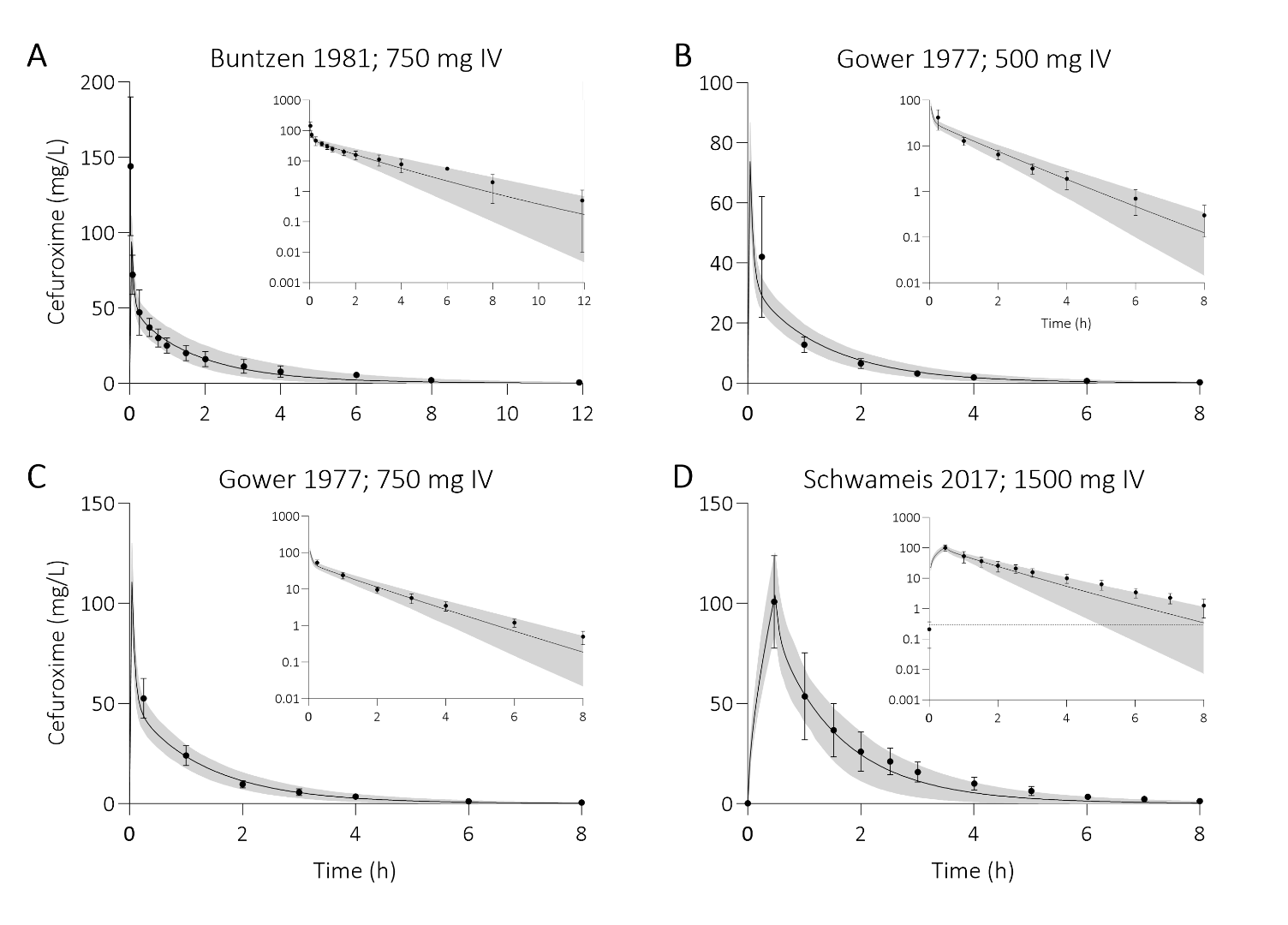


**Figure S3a.** **Visual predictive checks for cefuroxime in adults (single dose IV).** The solid line is the predicted mean of the simulated population and the shaded area represents the 5^th^ to 95^th^ percentile of the virtual population. Symbols are mean observed datapoints ± standard deviation, inserts are semi-log plots. ^23, 25, 28^ Dashed horizontal lines in the semi-log plots represent the reported lower limit of quantification (LLOQ) of the clinical studies Abbreviation: IV: intravenous.


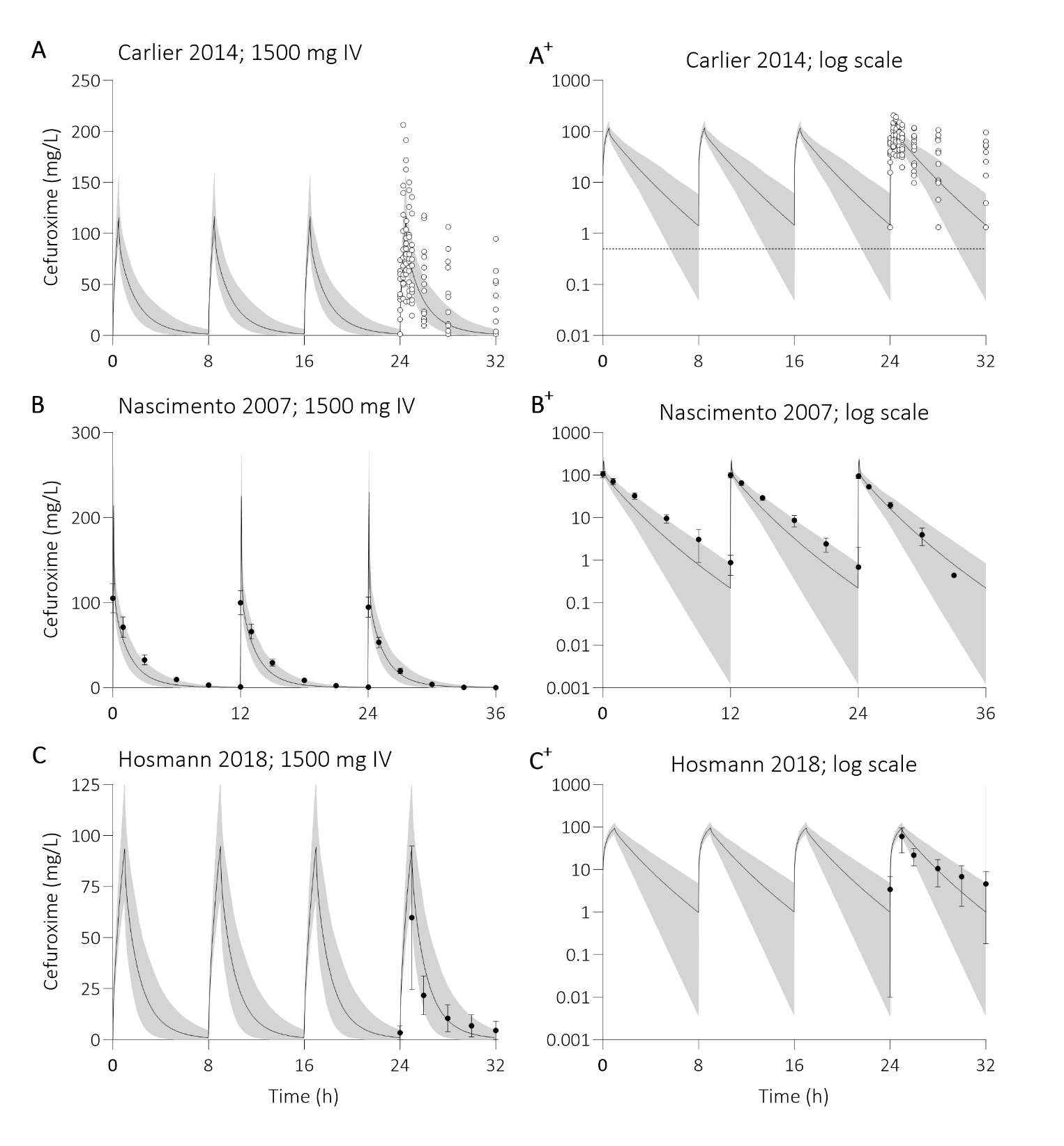


**Figure S3b.** **Visual predictive checks for cefuroxime in adults (multidose IV).** The solid line is the predicted mean of the simulated population and the shaded area represents the 5^th^ to 95^th^ percentile of the virtual population. Closed symbols are mean observed datapoints ± standard error of the mean (B, B^+^) or standard deviation (C, C^+^), open circles are individual datapoints. ^24, 26, 27^ A^+^, B^+^, and C^+^ are semi-log plots of A, B, and C, respectively. Dashed horizontal lines in the semi-log plots represent the reported lower limit of quantification (LLOQ) of the clinical studies. Abbreviation: IV: intravenous.


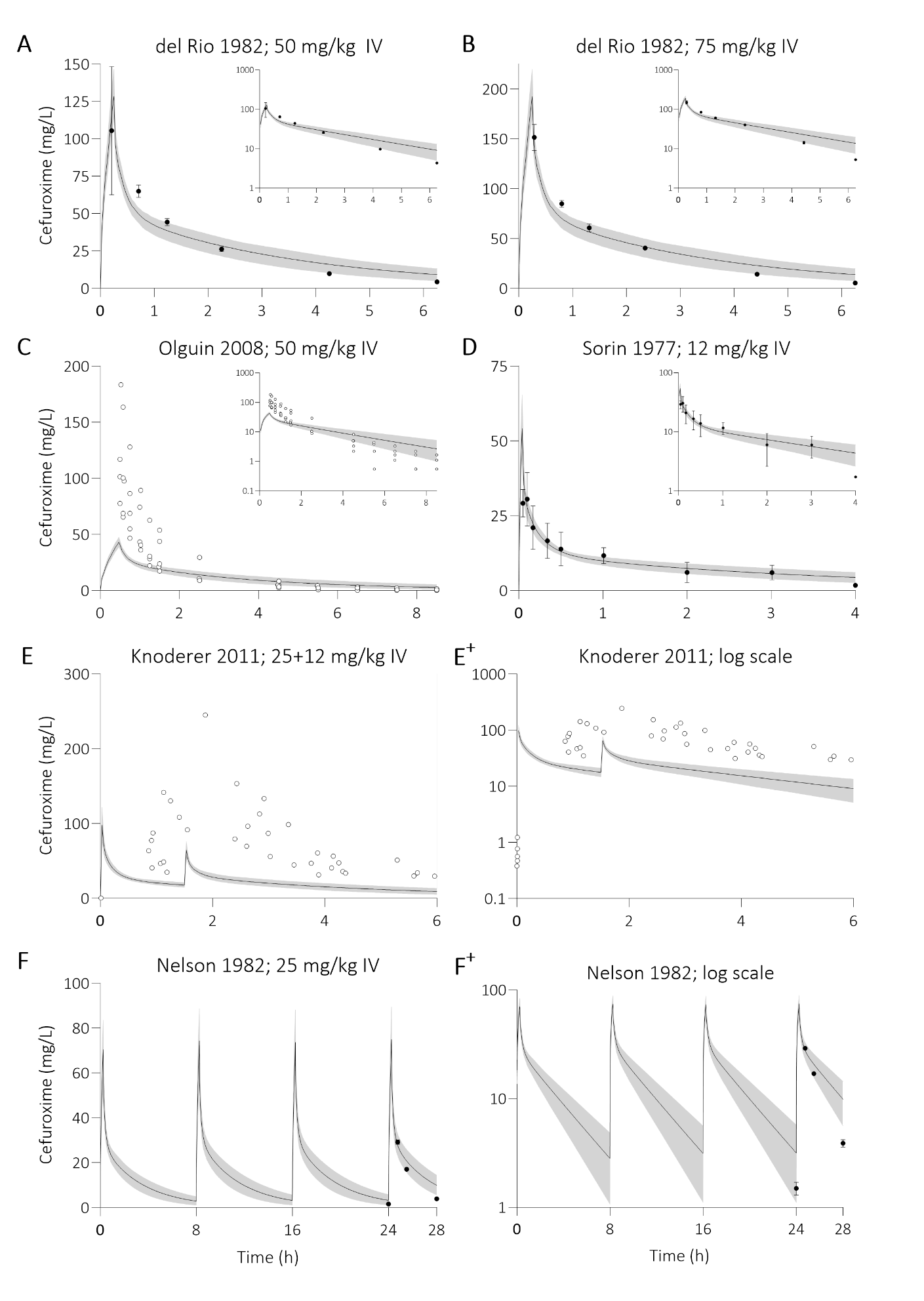


**Figure S3c.** **Visual predictive checks for cefuroxime in paediatrics (single and multidose IV).** The solid line is the predicted mean of the simulated population and the shaded area represents the 5^th^ to 95^th^ percentile of the virtual population. Closed symbols are mean observed datapoints ± standard deviation (A, B, D) or ± standard error of the mean (F, F^+^), open circles are individual datapoints. ^29-33^ Inserts are semi-log plots and E^+^ and F^+^ are semi-log plots of E and F, respectively. The data from Olguin are from children with reduced kidney function while the data from Knoderer et al. are from patients who underwent hypothermia. In both cases, reduced clearance is the result, which explains that the predicted exposure in lower than observed. Abbreviation: IV: intravenous.

## Ciprofloxacin


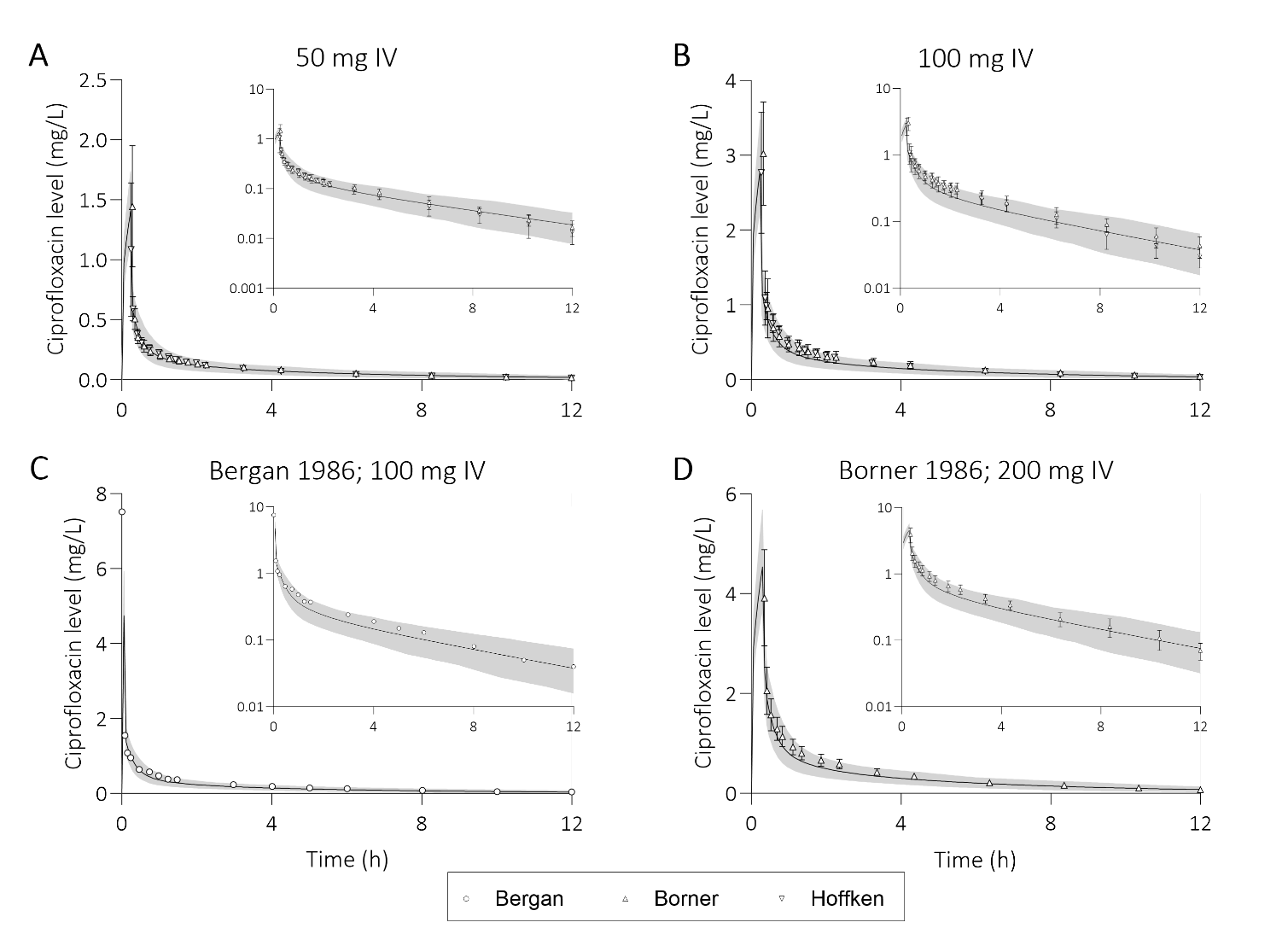


**Figure S4a.** **Visual predictive checks for ciprofloxacin in adults (single dose IV).** The solid line is the predicted mean of the simulated population and the shaded area represents the 5^th^ to 95^th^ percentile of the virtual population. Symbols are mean observed datapoints ± standard deviation, inserts are semi-log plots. ^34, 35, 37^ Abbreviation: IV: intravenous.


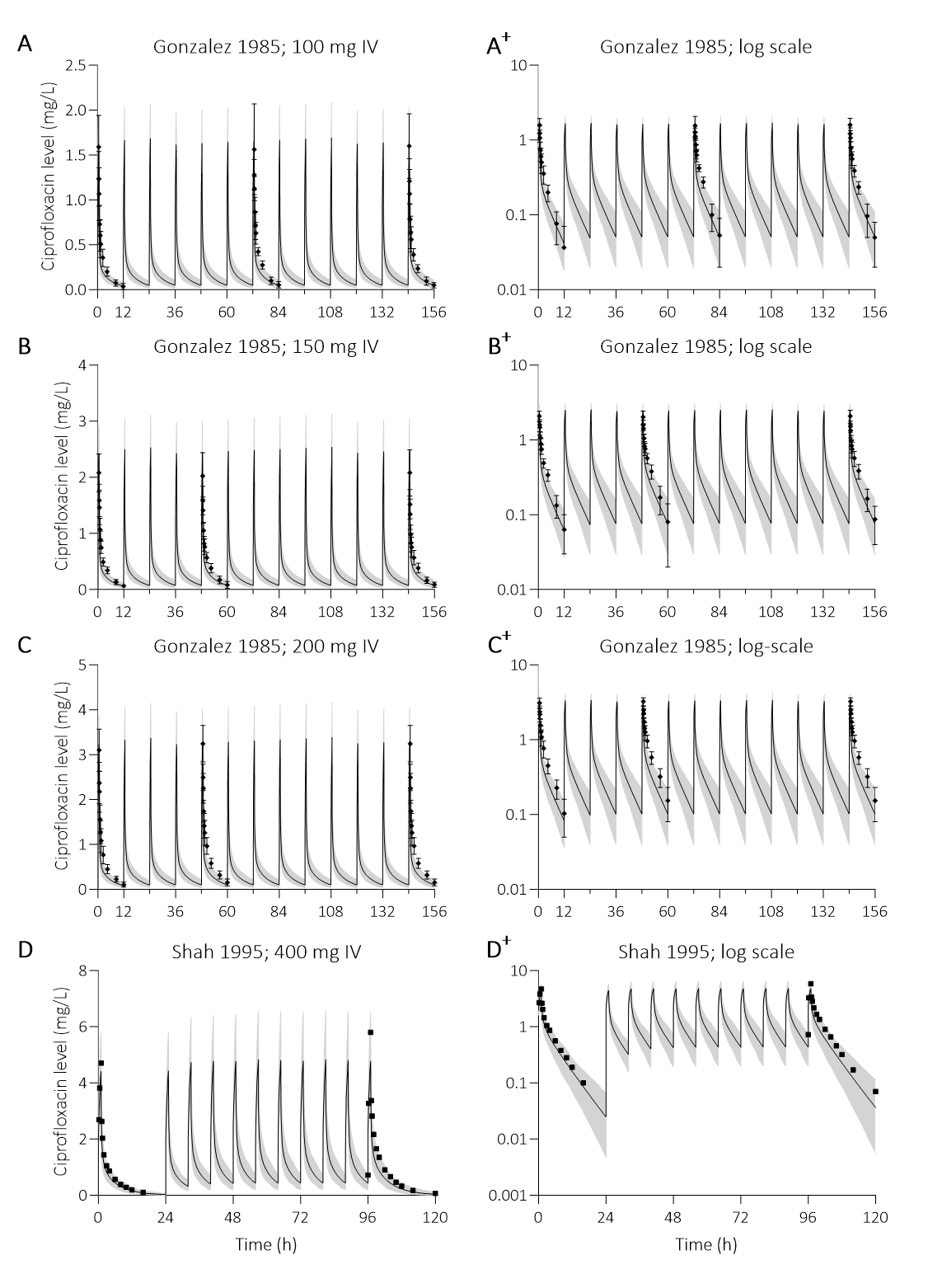


**Figure S4b.** **Visual predictive checks for ciprofloxacin in adults (multidose IV).** The solid line is the predicted mean of the simulated population and the shaded area represents the 5^th^ to 95^th^ percentile of the virtual population. Symbols are mean observed datapoints ± standard deviation. ^36, 39^ A^+^, B^+^, C^+^, and D^+^ are semi-log plots of A, B, C, and D, respectively. Abbreviation: IV: intravenous.

**Figure S4c.** **Visual predictive checks for ciprofloxacin in adults (single dose PO).** The solid line is the predicted mean of the simulated population and the shaded area represents the 5^th^ to 95^th^ percentile of the virtual population. Symbols are mean observed datapoints ± standard deviation, inserts are semi-log plots. ^34, 35, 37^ Abbreviation: PO: oral.


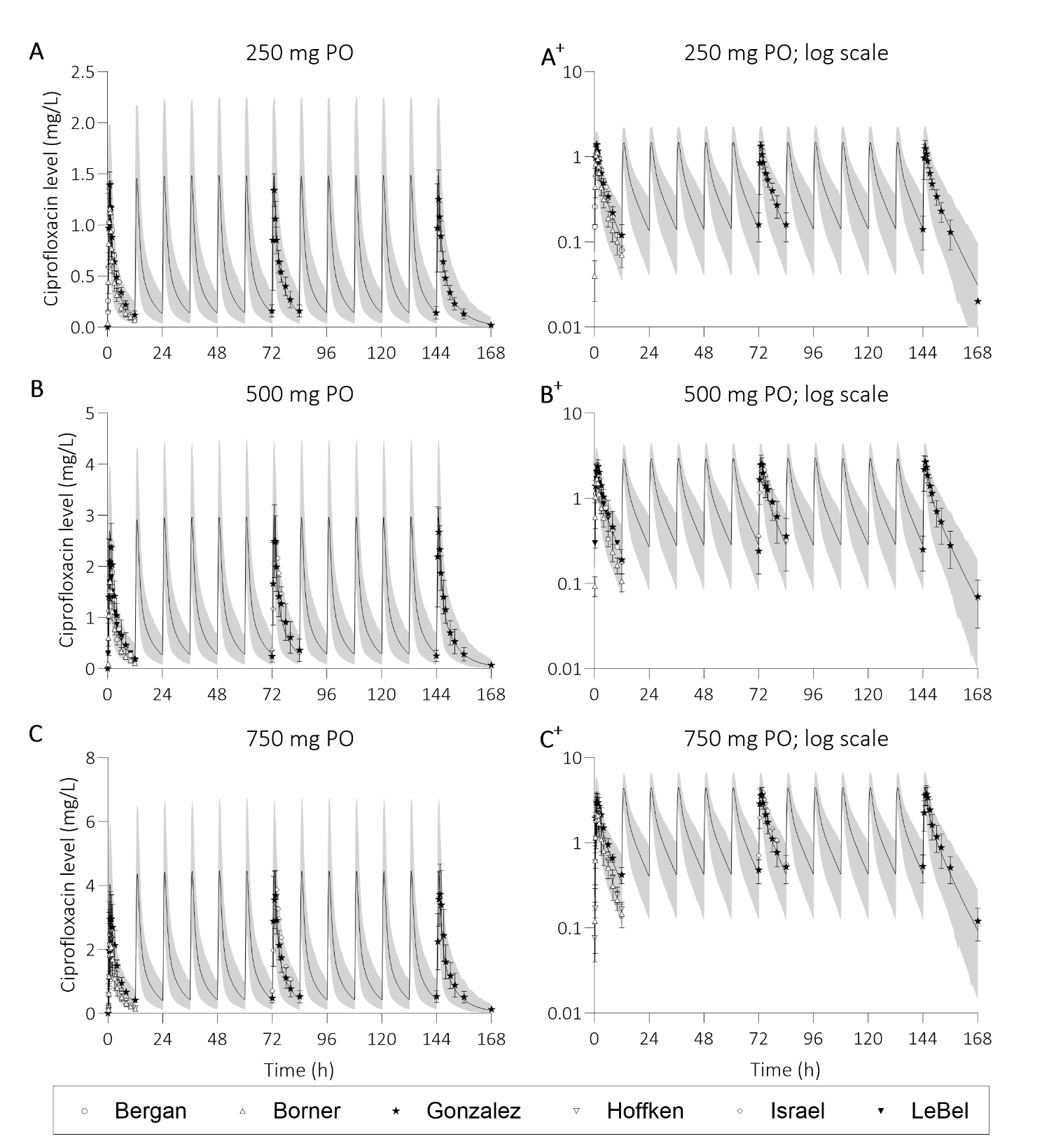


**Figure S4d.** **Visual predictive checks for ciprofloxacin in adults (multidose PO).** The solid line is the predicted mean of the simulated population and the shaded area represents the 5^th^ to 95^th^ percentile of the virtual population. Symbols are mean observed datapoints ± standard deviation. ^34, 35, 37, 40-42^ A^+^, B^+^, and C^+^ are semi-log plots of A, B, and C, respectively. Abbreviation: PO: oral.


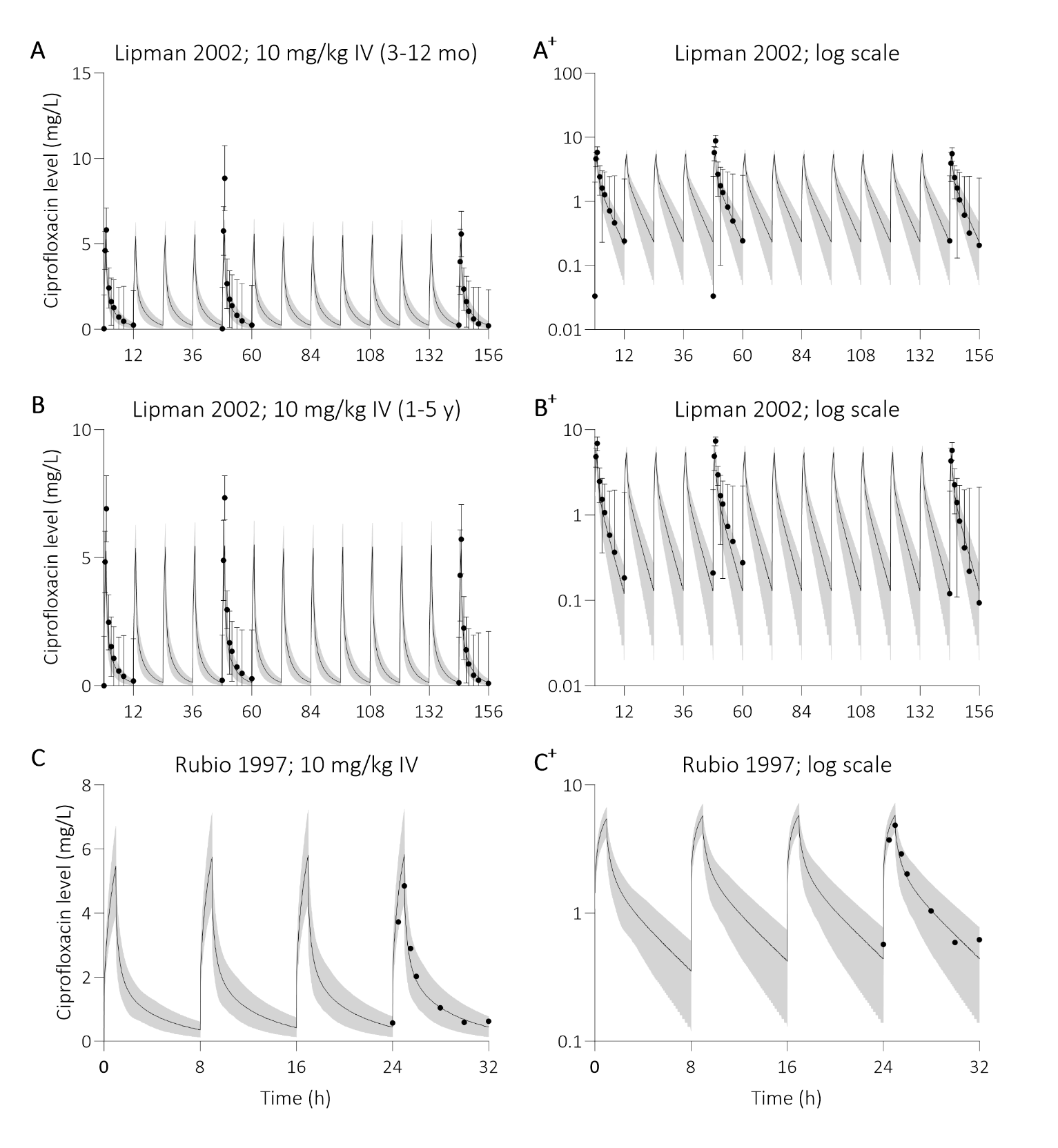


**Figure S4e.** **Visual predictive checks for ciprofloxacin in paediatrics (multidose IV).** The solid line is the predicted mean of the simulated population and the shaded area represents the 5^th^ to 95^th^ percentile of the virtual population. Symbols are mean observed datapoints ± standard deviation. ^43, 44^ A^+^, B^+^, and C^+^ are semi-log plots of A, B, and C, respectively. Abbreviation: IV: intravenous.


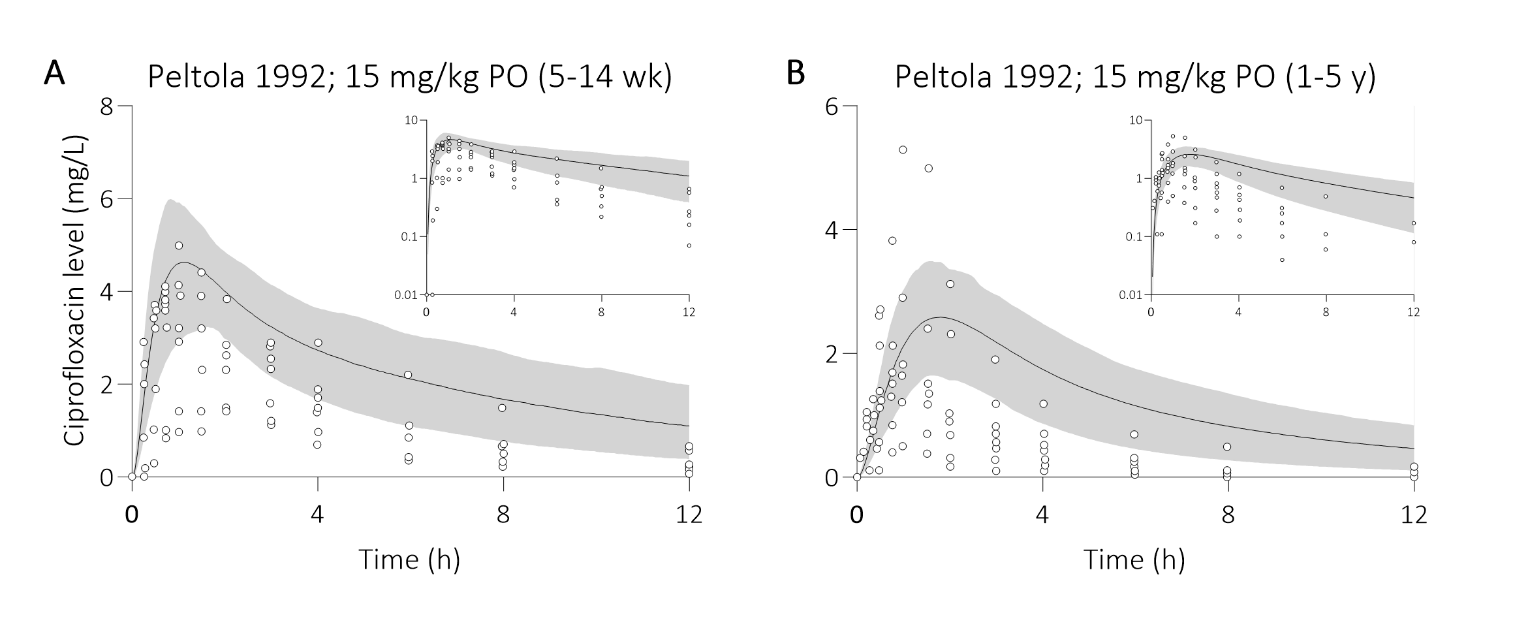


**Figure S4f.** **Visual predictive checks for ciprofloxacin in paediatrics (single dose PO).** The solid line is the predicted mean of the simulated population and the shaded area represents the 5^th^ to 95^th^ percentile of the virtual population. Symbols are individual observed datapoints, inserts are semi-log plots. ^46^ Abbreviation: PO: oral.


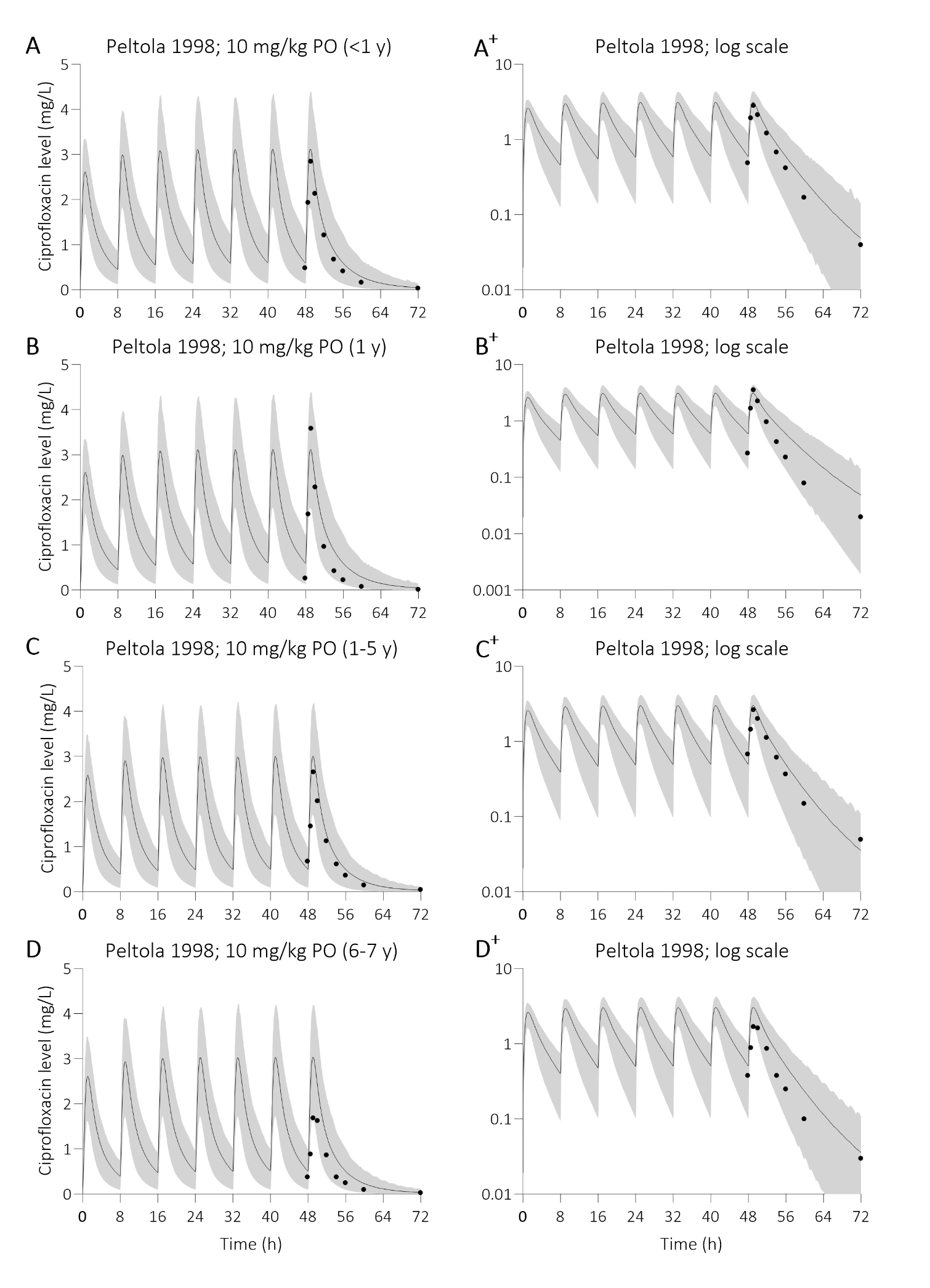


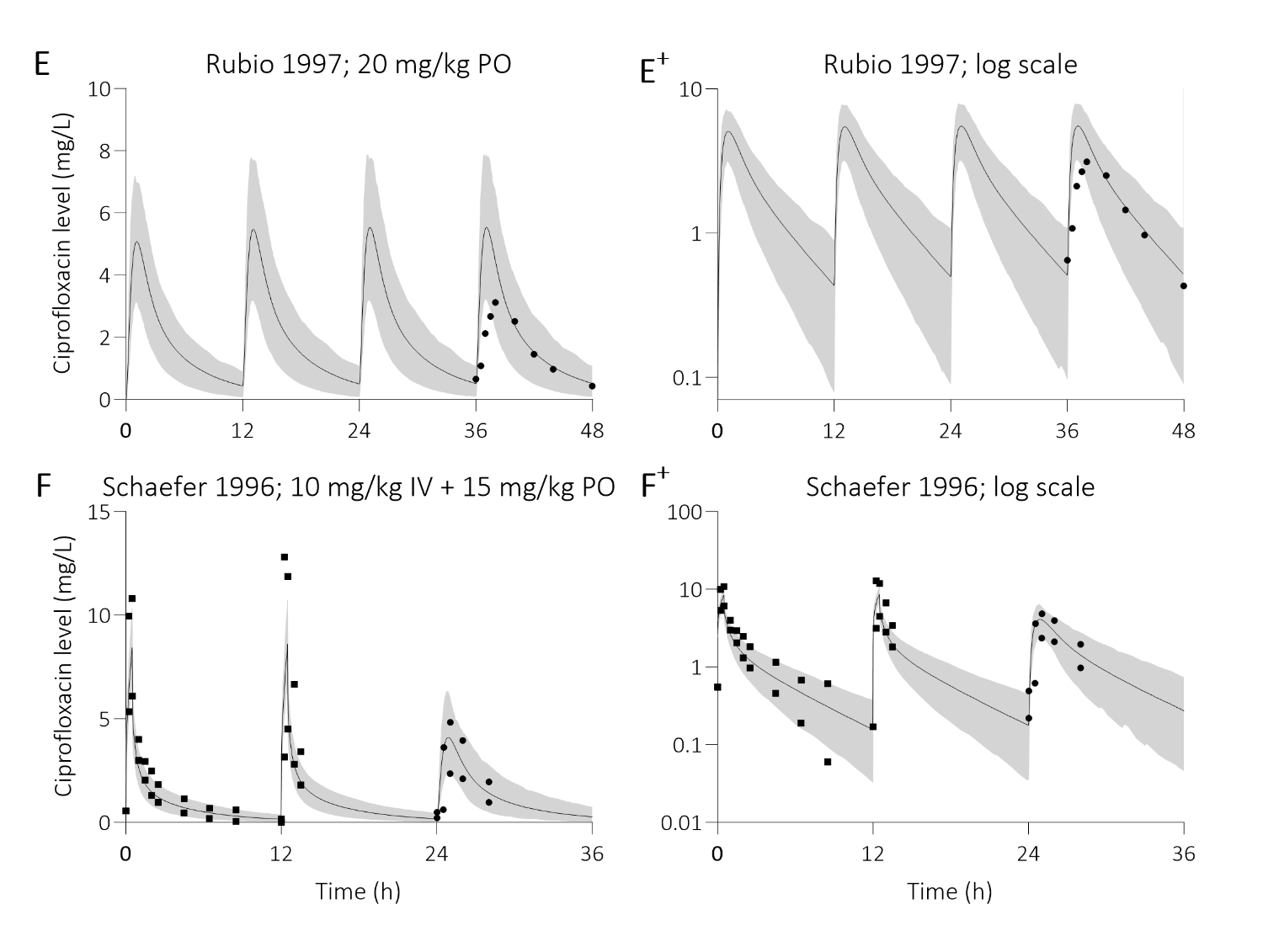


**Figure S4g.** **Visual predictive checks for ciprofloxacin in paediatrics (multidose PO).** The solid line is the predicted mean of the simulated population and the shaded area represents the 5^th^ to 95^th^ percentile of the virtual population. Circles are mean observed PO datapoints and squares are mean observed IV datapoints. ^44, 45, 47^ A^+^, B^+^, C^+^, D^+^, E^+^, and F^+^ are semi-log plots of A, B, C, D, E, and F, respectively. Abbreviations: IV: intravenous, PO: oral.

# 5. References

1. Abduljalil K, Ning J, Pansari A et al. Prediction of Maternal and Fetoplacental Concentrations of Cefazolin, Cefuroxime, and Amoxicillin during Pregnancy Using Bottom-Up Physiologically Based Pharmacokinetic Models. *Drug Metab Dispos* 2022; **50**: 386-400.

2. Salem F, Small BG, Johnson TN. Development and application of a pediatric mechanistic kidney model. *CPT Pharmacometrics Syst Pharmacol* 2022; **11**: 854-66.

3. Zhou L, Tong X, Sharma P et al. Physiologically based pharmacokinetic modelling to predict exposure differences in healthy volunteers and subjects with renal impairment: Ceftazidime case study. *Basic Clin Pharmacol Toxicol* 2019; **125**: 100-7.

4. van der Heijden JEM, Freriksen JJM, de Hoop-Sommen MA et al. Feasibility of a Pragmatic PBPK Modeling Approach: Towards Model-Informed Dosing in Pediatric Clinical Care. *Clin Pharmacokinet* 2022; **61**: 1705-17.

5. Arancibia A, Guttmann J, González G, González C. Absorption and disposition kinetics of amoxicillin in normal human subjects. *Antimicrob Agents Chemother* 1980; **17**: 199-202.

6. Paintaud G, Alván G, Dahl ML et al. Nonlinearity of amoxicillin absorption kinetics in human. *Eur J Clin Pharmacol* 1992; **43**: 283-8.

7. Witkowski G, Lode H, Höffken G, Koeppe P. Pharmacokinetic studies of amoxicillin, potassium clavulanate and their combination. *Eur J Clin Microbiol* 1982; **1**: 233-7.

8. Zarowny D, Ogilvie R, Tamblyn D et al. Pharmacokinetics of amoxicillin. *Clin Pharmacol Ther* 1974; **16**: 1045-51.

9. Ghim JL, Chin MC, Jung J et al. Pharmacokinetics and Pharmacodynamics of Tegoprazan Coadministered With Amoxicillin and Clarithromycin in Healthy Subjects. *J Clin Pharmacol* 2021; **61**: 913-22.

10. Liew KB, Loh GO, Tan YT, Peh KK. Randomized two-way cross-over bioequivalence study of two amoxicillin formulations and inter-ethnicity pharmacokinetic variation in healthy Malay volunteers. *Biomed Chromatogr* 2014; **28**: 1246-53.

11. Padoin C, Tod M, Brion N et al. Pharmacokinetics of amoxicillin coadministered with a saline-polyethylene glycol solution in healthy volunteers. *Biopharm Drug Dispos* 1995; **16**: 169-76.

12. Perveen S, Gauhar S, Yousuf RI et al. Evaluation of pharmacokinetic interactions of amoxicillin with ranitidine in healthy human volunteers of Karachi, Pakistan. *PLoS One* 2022; **17**: e0267791.

13. Ullah A, Azad MA, Sultana R et al. Bioequivalence evaluation of two capsule formulations of amoxicillin in healthy adult male bangladeshi volunteers: A single-dose, randomized, open-label, two-period crossover study. *Curr Ther Res Clin Exp* 2008; **69**: 504-13.

14. Schaad UB, Casey PA, Cooper DL. Single-dose pharmacokinetics of intravenous clavulanic acid with amoxicillin in pediatric patients. *Antimicrob Agents Chemother* 1983; **23**: 252-5.

15. Adrianzén Vargas MR, Danton MH, Javaid SM et al. Pharmacokinetics of intravenous flucloxacillin and amoxicillin in neonatal and infant cardiopulmonary bypass surgery. *Eur J Cardiothorac Surg* 2004; **25**: 256-60.

16. Rudoy RC, Goto N, Pettit D, Uemura H. Pharmacokinetics of intravenous amoxicillin in pediatric patients. *Antimicrob Agents Chemother* 1979; **15**: 628-9.

17. Fonseca W, Hoppu K, Rey LC et al. Comparing pharmacokinetics of amoxicillin given twice or three times per day to children older than 3 months with pneumonia. *Antimicrob Agents Chemother* 2003; **47**: 997-1001.

18. Ginsburg CM, McCracken GH, Jr., Thomas ML, Clahsen J. Comparative pharmacokinetics of amoxicillin and ampicillin in infants and children. *Pediatrics* 1979; **64**: 627-31.

19. Marks MI, Vose AD. Evaluation of amoxicillin therapy in ill children. *J Clin Pharmacol* 1978; **18**: 61-6.

20. Nelson JD, Kusmiesz H, Shelton S. Pharmacokinetics of potassium clavulanate in combination with amoxicillin in pediatric patients. *Antimicrob Agents Chemother* 1982; **21**: 681-2.

21. van Niekerk CH, van den Ende J, Hundt HK, Louw EA. Pharmacokinetic study of a paediatric formulation of amoxycillin and clavulanic acid in children. *Eur J Clin Pharmacol* 1985; **29**: 235-9.

22. Schaad UB, Casey PA, Ravenscroft AT. Pharmacokinetics of a syrup formulation of amoxycillin-potassium clavulanate in children. *J Antimicrob Chemother* 1986; **17**: 341-5.

23. Bundtzen RW, Toothaker RD, Nielson OS et al. Pharmacokinetics of cefuroxime in normal and impaired renal function: comparison of high-pressure liquid chromatography and microbiological assays. *Antimicrob Agents Chemother* 1981; **19**: 443-9.

24. Carlier M, Noë M, Roberts JA et al. Population pharmacokinetics and dosing simulations of cefuroxime in critically ill patients: non-standard dosing approaches are required to achieve therapeutic exposures. *J Antimicrob Chemother* 2014; **69**: 2797-803.

25. Gower PE, Dash CH. The pharmacokinetics of cefuroxime after intravenous injection. *Eur J Clin Pharmacol* 1977; **12**: 221-7.

26. Hosmann A, Ritscher LC, Burgmann H et al. Concentrations of Cefuroxime in Brain Tissue of Neurointensive Care Patients. *Antimicrob Agents Chemother* 2018; **62**.

27. Nascimento JW, Carmona MJ, Strabelli TM et al. Perioperative cefuroxime pharmacokinetics in cardiac surgery. *Clinics (Sao Paulo)* 2007; **62**: 257-60.

28. Schwameis R, Syré S, Marhofer D et al. Pharmacokinetics of Cefuroxime in Synovial Fluid. *Antimicrob Agents Chemother* 2017; **61**.

29. del Rio Mde L, Chrane DF, Shelton S et al. Pharmacokinetics of cefuroxime in infants and children with bacterial meningitis. *Antimicrob Agents Chemother* 1982; **22**: 990-4.

30. Knoderer CA, Saft SA, Walker SG et al. Cefuroxime pharmacokinetics in pediatric cardiovascular surgery patients undergoing cardiopulmonary bypass. *J Cardiothorac Vasc Anesth* 2011; **25**: 425-30.

31. Nelson JD, Kusmiesz H, Shelton S. Cefuroxime therapy for pneumonia in infants and children. *Pediatr Infect Dis* 1982; **1**: 159-63.

32. Olguín HJ, Asseff IL, Vieyra AC et al. Effect of severity disease on the pharmacokinetics of cefuroxime in children with multiple organ system failure. *Biol Pharm Bull* 2008; **31**: 316-20.

33. Sorin M, Ghnassia JC, Demerleire F, Saudubray JM. Pharmacokinetic and clinical study of cefuroxime in infants. *Proc R Soc Med* 1977; **70**: 175-8.

34. Bergan T, Thorsteinsson SB, Kolstad IM, Johnsen S. Pharmacokinetics of ciprofloxacin after intravenous and increasing oral doses. *Eur J Clin Microbiol* 1986; **5**: 187-92.

35. Borner K, Höffken G, Lode H et al. Pharmacokinetics of ciprofloxacin in healthy volunteers after oral and intravenous administration. *Eur J Clin Microbiol* 1986; **5**: 179-86.

36. Gonzalez MA, Moranchel AH, Duran S et al. Multiple-dose pharmacokinetics of ciprofloxacin administered intravenously to normal volunteers. *Antimicrob Agents Chemother* 1985; **28**: 235-9.

37. Höffken G, Lode H, Prinzing C et al. Pharmacokinetics of ciprofloxacin after oral and parenteral administration. *Antimicrob Agents Chemother* 1985; **27**: 375-9.

38. Lipman J, Scribante J, Gous AG et al. Pharmacokinetic profiles of high-dose intravenous ciprofloxacin in severe sepsis. The Baragwanath Ciprofloxacin Study Group. *Antimicrob Agents Chemother* 1998; **42**: 2235-9.

39. Shah A, Lettieri J, Nix D et al. Pharmacokinetics of high-dose intravenous ciprofloxacin in young and elderly and in male and female subjects. *Antimicrob Agents Chemother* 1995; **39**: 1003-6.

40. Gonzalez MA, Uribe F, Moisen SD et al. Multiple-dose pharmacokinetics and safety of ciprofloxacin in normal volunteers. *Antimicrob Agents Chemother* 1984; **26**: 741-4.

41. Israel D, Gillum JG, Turik M et al. Pharmacokinetics and serum bactericidal titers of ciprofloxacin and ofloxacin following multiple oral doses in healthy volunteers. *Antimicrob Agents Chemother* 1993; **37**: 2193-9.

42. LeBel M, Barbeau G, Bergeron MG et al. Pharmacokinetics of ciprofloxacin in elderly subjects. *Pharmacotherapy* 1986; **6**: 87-91.

43. Lipman J, Gous AG, Mathivha LR et al. Ciprofloxacin pharmacokinetic profiles in paediatric sepsis: how much ciprofloxacin is enough? *Intensive Care Med* 2002; **28**: 493-500.

44. Rubio TT, Miles MV, Lettieri JT et al. Pharmacokinetic disposition of sequential intravenous/oral ciprofloxacin in pediatric cystic fibrosis patients with acute pulmonary exacerbation. *Pediatr Infect Dis J* 1997; **16**: 112-7; discussion 23-6.

45. Schaefer HG, Stass H, Wedgwood J et al. Pharmacokinetics of ciprofloxacin in pediatric cystic fibrosis patients. *Antimicrob Agents Chemother* 1996; **40**: 29-34.

46. Peltola H, Väärälä M, Renkonen OV, Neuvonen PJ. Pharmacokinetics of single-dose oral ciprofloxacin in infants and small children. *Antimicrob Agents Chemother* 1992; **36**: 1086-90.

47. Peltola H, Ukkonen P, Saxén H, Stass H. Single-dose and steady-state pharmacokinetics of a new oral suspension of ciprofloxacin in children. *Pediatrics* 1998; **101**: 658-62.
